# Non-Musculoskeletal Benefits of Vitamin D beyond the Musculoskeletal System

**DOI:** 10.3390/ijms22042128

**Published:** 2021-02-21

**Authors:** Sicheng Zhang, Duane D. Miller, Wei Li

**Affiliations:** Department of Pharmaceutical Sciences, College of Pharmacy, The University of Tennessee Health Science Center, Memphis, TN 38163, USA; szhang71@uthsc.edu (S.Z.); dmiller@uthsc.edu (D.D.M.)

**Keywords:** vitamin D, musculoskeletal, nonmusculoskeletal, 25-hydroxyvitamin D (25(OH)D), 1α,25-dihydroxyvitamin D (1α,25(OH)_2_D), sunlight

## Abstract

Vitamin D, a fat-soluble prohormone, is endogenously synthesized in response to sunlight or taken from dietary supplements. Since vitamin D receptors are present in most tissues and cells in the body, the mounting understanding of the role of vitamin D in humans indicates that it does not only play an important role in the musculoskeletal system, but has beneficial effects elsewhere as well. This review summarizes the metabolism of vitamin D, the research regarding the possible risk factors leading to vitamin D deficiency, and the relationships between vitamin D deficiency and numerous illnesses, including rickets, osteoporosis and osteomalacia, muscle weakness and falls, autoimmune disorders, infectious diseases, cardiovascular diseases (CVDs), cancers, and neurological disorders. The system-wide effects of vitamin D and the mechanisms of the diseases are also discussed. Although accumulating evidence supports associations of vitamin D deficiency with physical and mental disorders and beneficial effects of vitamin D with health maintenance and disease prevention, there continue to be controversies over the beneficial effects of vitamin D. Thus, more well-designed and statistically powered trials are required to enable the assessment of vitamin D’s role in optimizing health and preventing disease.

## 1. Introduction

In the mid-17th century, most North-European children who lived in heavily industrialized, polluted cities developed rickets, a severe bone-deforming disease that was characterized by bowed legs, knobby projections of the ribcage, bending of the spine, a large forehead, weak and toneless muscles, and stunted growth. [1,2,3] Rickets was effectively eradicated in the United States and Europe during the 1930s by the fortification of milk with vitamin D [4]. Vitamin D is a lipid-soluble vitamin, which, however, acts more like a hormone because it can not only be ingested from food and supplements but can also be produced endogenously in humans. The recent interest in vitamin D has been driven by the mounting recognition of its non-musculoskeletal functions beyond its role in skeletal health, including calcium homeostasis and bone metabolism. Vitamin D deficiency is now recognized as a global health issue that afflicts more than half of the world’s population [5,6], and its potential impact on human health is an area of expanding research. In this review, we summarize the factors and consequences of vitamin D deficiency, and the roles of vitamin D in musculoskeletal and non-musculoskeletal health. Despite the positive effects found in cell and murine studies, however, several findings from randomized controlled trials (RCTs) of vitamin D supplementation and nonmusculoskeletal health outcomes are inconsistent, and thus more well-designed and statistically powered trials are required to settle the controversy.

## 2. Biology and Metabolism

Vitamins D_2_ (ergocalciferol) and D_3_ (cholecalciferol) are the two major forms of vitamin D. Vitamin D_3_ can be obtained directly from animal sources, but it is mainly synthesized by the skin naturally after exposure to ultraviolet (UV) light [7]. The ring-opened compound, previtamin D_3_, is generated from irradiation of 7-dehydrocholesterol (7-DHC) in the epidermis of the skin, which is followed by thermal isomerization to form vitamin D_3_. A similar process takes place in fungi, where UV irradiation leads to the formation of vitamin D_2_ from ergosterol.

### 2.1. Classical Pathway of Vitamin D Activation

**Figure 1:**
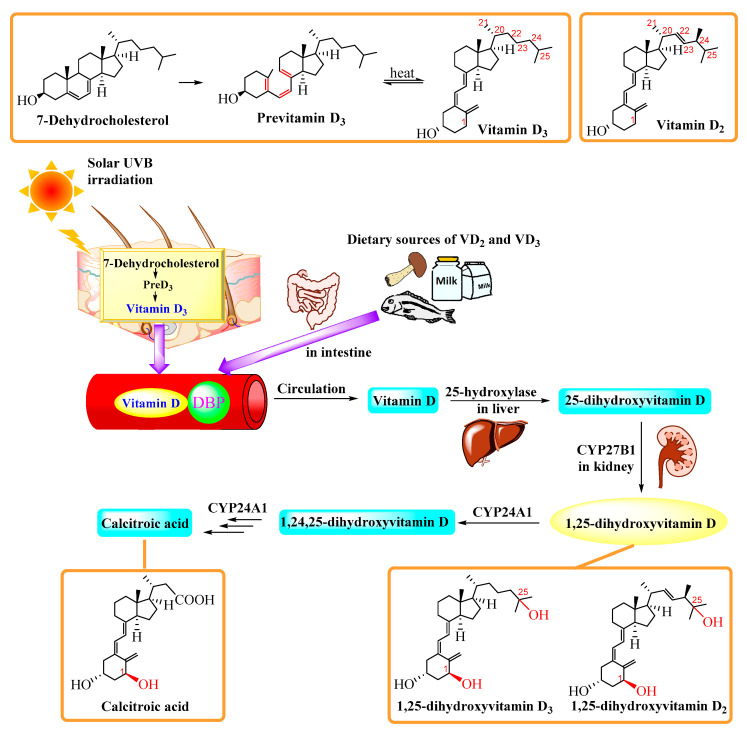
The photoproduction and metabolism of vitamin D. Vitamin D_3_ is produced in the skin via a two-step process from the vitamin D substrate 7-DHC, which is converted to previtamin D_3_ upon exposure to solar ultraviolet B (UVB) radiation, followed by thermal conversion to vitamin D_3_. Vitamin D_2_ and vitamin D_3_ from dietary sources together with endogenous vitamin D_3_ are diffused into the circulatory system and bound to vitamin D binding protein (DBP). Vitamin D (hereafter “D” represents D_2_ or D_3_) is firstly converted by 25-hydroxylase to 25(OH)D primarily, but not exclusively, in the liver. 25(OH)D is biologically inactive, and it must be further hydroxylated by 1α-hydroxylase (CYP27B1) into the active form 1α,25(OH)_2_D in the kidney or other targeted cells and tissues. This active form can induce the expression of the enzyme 24-hydroxylase (24-OHase) upon completion of the task. The 24-OHAse enhances the catabolism of 1α,25(OH)_2_D into 1,24,25-hydroxyvitamin D, which can then be successively oxidized into the biologically inert calcitroic acid.

Vitamin D that comes from the skin or dietary sources is biologically inert, and two separate hydroxylations are required for full hormonal potency in the human body. The first occurs in the liver and converts vitamin D to 25(OH)D or calcidiol by a 25-hydroxylase such as CYP3A4 [8], CYP2R1 [9], CYP2J3 [10], or CYP27A1 [11]. A further hydroxylation occurs primarily in the proximal tubule of the kidney with the aid of mitochondrial 1α-hydroxylase (CYP27B1) to give 1α,25(OH)_2_D, or calcitriol, the physiologically active form of vitamin D [12]. Vitamin D acts through vitamin D receptor (VDR), a specific nuclear binding receptor which is expressed in most tissues throughout the body. Activation of the VDR by its ligand 1α,25(OH)_2_D leads to attachment of this transcription complex to the respective vitamin D responsive element (VDRE) of the DNA [13]. It was initially thought that the two forms of vitamin D follow the same metabolic pathway. However, minor differences in the structures of side chains between vitamin D_2_ and D_3_ result in differences in the hydroxylation site and lead to the production of unique biologically active metabolites. After a series of oxidations and hydroxylations, both the two forms of vitamin D give rise to calcitroic acid (Figure 1).

### 2.2. Non-Classical Pathway of Vitamin D Activation

Recently, an alternative pathway for vitamin D activation by CYP11A1 has been established. The favored initial hydroxylation occurs at C20, with 20(OH)D being the major metabolite [14,15]. Other identified sites of hydroxylation of the vitamin D_3_ side chain by CYP11A1 are C17, C22, and C23, and at least 10 metabolites, such as 17(OH)D_3_, 22(OH)D_3_, 17,20(OH)_2_D_3_, 20,22(OH)_2_D_3_, 20,23(OH)_2_D_3_, and 17,20,23(OH)_3_D_3_, are produced [16,17]. 20(OH)D_3_ and its hydroxymetabolites inhibit DNA synthesis and colony formation, induce cell cycle arrest, and stimulate the differentiation of keratinocytes with a potency comparable to or better than that of 1,25(OH)_2_D_3_ [18,19,20,21]. These hydroxymetabolites also show cell lineage-dependent anti-cancer properties [22,23,24,25,26]. It has been reported that 20(OH)D_3_ and 20,23(OH)_2_D_3_ enhance defense mechanisms against UVB-induced oxidative stress and DNA damage in cultured human keratinocytes [27] and murine skin in vivo [28]. As for vitamin D_2_, in addition to 20(OH)D_2_, another major product was identified as 17,20(OH)_2_D_2_, along with some lesser production of 17,20,24(OH)_3_D_2_ [15]. Studies on 20(OH)D_2_ have demonstrated that it can also inhibit proliferation and induce the differentiation of keratinocytes [29], and inhibit the proliferation and behavior of normal and malignant melanocytes in a similar manner to 1,25(OH)_2_D_3_ [30]. Noteworthily, several CYP11A1-derived D_3_ hydroxymetabolites, such as 20(OH)D_3_, 20(OH)D_2_, and 20,23(OH)_2_D_3_, lack toxicities and calcemic effects at very high doses (3–60 µg/kg) in mice, in contrast to 1,25(OH)_2_D_3_ and 25(OH)D_3_ [25,30,31].

There has been much debate concerning the relative abilities of vitamin D_2_ and D_3_ to raise vitamin D status. Vitamin D_2_ was first produced in the early 1920s, and the process was patented and licensed to pharmaceutical companies, which led to the development of a medicinal preparation of vitamin D_2_. Pharmacopoeias have officially regarded these two forms as equivalent and interchangeable, yet disagreement exists. Some reports indicate that vitamin D_3_, which has a higher binding affinity to vitamin D binding protein, is two to three times more effective at increasing blood levels of 25(OH)D than the equivalent dose of vitamin D_2_ [32], and several studies suggest that vitamin D_2_ may indeed have a more rapid turnover rate in the serum than vitamin D_3_, though the difference may be inconsequential with daily dosing of vitamin D [33,34,35,36]. Although many major prescription forms of vitamin D are actually vitamin D_2_ rather than vitamin D_3_, most companies are now reformulating their products to contain vitamin D in the D_3_ form, due to the growing belief that vitamin D_3_ is the better form to be used. However, firm conclusions about any different effects of these two forms of vitamin D cannot be drawn and more research is needed.

## 3. Definition of Vitamin D Deficiency

The definition of vitamin D deficiency in the past by the clinical diagnosis of nutritional rickets has expanded to a definition based on the serum concentration of 25(OH)D. Although the 25(OH)D metabolite has no physiological function, it is widely used as an indicator to determine a person’s vitamin D status because it reflects vitamin D supply from dietary exposure and endogenous synthesis, but also because it is the major circulating form of vitamin D with a long half-life (in the circulation) of 2–3 weeks and it is not under tight homeostatic control. In contrast, 1α,25(OH)_2_D, as the active form, is not a suitable indicator because it is homeostatically regulated and has a short half-life (<4h) [37,38,39,40]. Measurement of serum 25(OH)D includes automated enzyme-linked immunosorbent assay, radioimmunoassay, automated immunoassays, high performance liquid chromatography (HPLC), and liquid chromatography coupled with tandem mass spectroscopy (LC-MS/MS). Currently, HPLC with MS/MS detection has been established as the gold standard for serum 25(OH)D level testing [41].

There is yet no consensus on optimal serum 25(OH)D levels, since different organizations and institutions have their own definitions of vitamin D status and recommendation for supplementation [42], leading to difficulty in creating an accurate definition of vitamin D deficiency. Despite the controversy, serum levels of 25(OH)D below 20 ng/mL (50 nmol/L) should be avoided, since they may cause increases in parathyroid hormone (PTH) [43]. An increase in PTH mediates the mobilization of calcium from bone, resulting in a reduction of bone mass and consequently an increased number of fractures [44].

## 4. Factors Leading to Vitamin D Deficiency

Several factors may contribute to the prevalence of vitamin D deficiency and the resurgence of rickets in our modern society, including variations in sun exposure; age; obesity; and several chronic illnesses, such as kidney, liver, and celiac diseases [45].

### 4.1. Exposure to Sunlight

Vitamin D_3_ is also called the “sunshine vitamin” because the main source of vitamin D_3_ for most humans is exposure to sunlight [46,47]. Ultraviolet radiation from the sun is categorized into three types according to wavelength: UVA (315–400 nm), UVB (280–315 nm), and UVC (100–280 nm). For cutaneous vitamin D_3_ synthesis, the action spectrum for UV-induced conversion of 7-DHC to previtamin D_3_ in human skin falls within the UVB range [7], indicating a maximum at about 297 nm with essentially no production above 315 nm. Although recently there have arisen findings casting doubt [48] on the accuracy of this universally recognized action spectrum, it represents a milestone in vitamin D research.

Any barrier that prevents the transmission of solar UVB radiation to the earth’s surface or anything that interferes with the penetration of UVB radiation into the skin may significantly reduce vitamin D_3_ production. For example, a 12.5% decrease in the atmospheric ozone value from 320 to 280 DU (DU = Dobson unit) at a fixed southern hemisphere site (27.5°S, 151°E) in clear sky conditions results in an approximate 15% increase in the monthly amount of vitamin D_3_-effective UVB radiation that reaches the earth’s surface [49]. Additionally, UVB can be absorbed, scattered, or reflected by various additional substances as it travels through the atmosphere, including oxygen and nitrogen, aerosols, water vapor, particulate pollutants, and clouds. For example, thick clouds are found to maintain only 1% of the surface UVB radiation of clear sky levels [50]. Black carbon particulates from biomass and fossil fuel combustion result in local reductions of UVB radiation by approximately 5% in typical urban environments [51] and up to 81% in the rainforests of Brazil [52].

Solar zenith angle (SZA), an angle between the local vertical (zenith) and a line from the observer to the sun, is another key factor influencing UVB radiation. An appropriate SZA is required for UVB to penetrate the non-polluted ozone. In general, the UVB radiation level increases at a smaller SZA and reaches a maximum at mid-day in the summer [53]. UVB radiation can hardly reach the earth’s surface at latitudes above 35°N and below 35°S during the winter months, which produces an almost complete cessation of cutaneous vitamin D synthesis [54]. For example, in Berlin, Germany (latitude 52.5°N) or Amsterdam, Netherlands (latitude 52.4°N), vitamin D_3_ is not able to be produced between October and April [55].

### 4.2. Cutaneous Factors

Prior to triggering vitamin D synthesis from 7-DHC, several factors further attenuate the UVB radiation level. Purdah and cultural coverings limit sunlight exposure and cutaneous vitamin D synthesis, which explains why both children and adults are commonly at high risk of vitamin D deficiency even in the sunniest areas of the world [56]. Melanin in the epidermis of darkly pigmented skin acts as an effective natural sunscreen, which is extremely efficient at absorbing UVB radiation and thus reduces vitamin D synthesis. Compared to individuals with lower concentrations of melanin, those with darkly pigmented skin need longer UV exposure times to generate the equivalent amount of vitamin D [57]. Observational studies [58,59,60] reported that individuals with higher melanization in the skin have poorer vitamin D status than those with lighter skin at comparable latitude. The explanation for these observations is that the capability of melanin to absorb UVB energy attenuates the final UVB dose reaching epidermal 7-DHC, which thus inhibits the previtamin D_3_ production [61]. On the other hand, several studies [59,62] showed that skin pigmentation does not influence the synthesis of vitamin D and 25(OH)D. Although it was difficult to integrate the contradictory evidence, a systematic review in 2015 [63] concluded that studies reporting an inhibitory effect of melanin were more convincing than those that observed no influence. However, a very recent study [64] indicated that compared to erythema, melanin offered limited inhibition of vitamin D_3_ production.

In addition, there are controversies about the association between vitamin D deficiency and sunscreen application. Several experimental studies [65,66,67,68] were in line with the expectation that sunscreen use abrogated the vitamin D_3_ or 25(OH)D production after exposure to nonsolar UV radiation. In contrast, a holiday study [69] on 79 healthy Polish volunteers (most with Fitzpatrick skin type II and III) showed that considerable production of vitamin D still occurred in the sunscreen-use group (sun protection factor 15, ≥ 2 mg cm^−2^) compared to that of the discretionary sunscreen-use group, suggesting that typical sunscreen use does not cause vitamin D insufficiency in healthy people with lighter skin types [42]. Overall, the risk of vitamin D deficiency resulting from sunscreen use might be lower than the risks resulting from other behaviors, such as staying in the shade and wearing protective clothing, and it is unlikely to be outweighed by the benefits for skin cancer prevention.

Cutaneous vitamin D_3_ synthesis is also influenced by 7-DHC levels. Post-burn scar tissue only contains as much as 42.5% of the 7-DHC typically found in normal skin, and burn patients often develop progressive vitamin D deficiency if they lack supplementation [70]. Advancing age decreases cutaneous 7-DHC as well. A 70-year-old has only 25% of the 7-DHC that a vicenarian does, and thus has a 75% reduced capacity to make vitamin D in the skin [71].

### 4.3. Bioavailability of Vitamin D after Oral Ingestion or Cutaneous Synthesis

#### 4.3.1. Bioavailability Decrease

##### Fat Malabsorption

Vitamin D is lipid soluble; therefore, it requires some dietary fat in the gut for absorption [72]. After being absorbed with long-chain triglycerides in the small intestine, ingested vitamin D is incorporated into chylomicrons within the enterocytes and then transported through the lymph system into the systemic circulation [73,74]. Any intestinal malabsorption disorder may impair the absorption of vitamin D due to a decreased ability to absorb lipids. Absorption decreased by 50%, > 72%, and > 82% for the oral dose in patients with celiac disease, biliary obstruction absorption, and chronic pancreatitis, respectively. Impaired vitamin D absorption was positively associated with the steatorrhea in each case. Furthermore, other conditions, such as liver failure, cystic fibrosis, Crohn’s disease (CD), and gastric bypass, cause impaired vitamin D absorption. This disorder can also be found in individuals who take bile acid-binding medications such as cholestyramine and colestipol for hypercholesterolemia [3,72].

##### Obesity

As discussed above, vitamin D is readily taken up by adipose tissues. Vitamin D can be stored in these tissues for subsequent release and metabolism in case production is reduced, such as during the winter months [75]. However, there seems to be an inverse correlation between adipose tissue levels and vitamin D status. Several studies have shown that obese individuals tend to have lower serum levels of vitamin D_3_ and 25(OH)D_3_ than those with normal weights [76,77,78]. Evaluation of serum vitamin D_3_ levels 24 h after whole-body irradiation showed that the increase in vitamin D_3_ was 57% lower in obese individuals with body mass index (BMI; in kg/m^2^) ≥ 30 than age-matched lean control subjects (BMI ≤ 25). The study also found that BMI was inversely associated with peak plasma concentrations of vitamin D_2_ after intake of oral doses of 50,000 IU (IU = international unit, 1 IU = 0.025 micrograms) of vitamin D_2_ [79]. Thus, obesity correlates with vitamin D deficiency and decreased vitamin D bioavailability. This is likely secondary to the sequestration of vitamin D into larger body fat compartments [80].

#### 4.3.2. Increases in the Metabolism of Vitamin D

Metabolism of 25(OH)D and 1α,25(OH)_2_D is primarily mediated by two cytochrome P450 enzymes. CYP24A1, which acts as 24-hydroxylase, initiates the breakdown of 25(OH)D and 1α,25(OH)_2_D in the kidney, and to a lesser extent, other tissues, whereas CYP3A4 mediates their metabolism in the liver and small intestine [81]. The combined activity of these two enzymes is an important factor in determining the circulating concentrations of 25(OH)D and 1α,25(OH)_2_D [82]. Long-term use of certain medications, including anticonvulsants, glucocorticoids, phenobarbital, phenytoin, carbamazepine, rifampicin, and antiretrovirals, causes upregulation of CYP3A4. This enhances the metabolism of 25(OH)D and 1α,25(OH)_2_D and leads to decreased levels of 25(OH)D and 1α,25(OH)_2_D [83,84,85].

##### Liver Disease

The liver, which is the site for conversion of vitamin D to 25(OH)D, plays a critical role in the maintenance of vitamin D status. Hepatobiliary disease is often related to vitamin D deficiency [86]. A decrease in the intestinal availability of bile salts in cholestatic liver disease leads to malabsorption of fat-soluble vitamins such as vitamin D. Data from one study showed that following a single oral dose of 1000 IU/ kg, the rise in serum vitamin D_2_ levels in six children with chronic cholestasis since infancy (mean age 12.1 years) was 98.7% lower than in controls [87]. The low serum 25(OH)D levels in severe parenchymal liver disease are mainly due to vitamin D malabsorption and reduced capacity for 25-hydroxylation. A study of 100 patients with noncholestatic chronic liver disease found serum 25(OH)D levels < 50 nmol/L in 86.3% of cirrhotic patients compared with only 49.0% of noncirrhotic controls [88]. As the primary carrier protein for vitamin D, 25(OH)D, and 1α,25(OH)_2_D in circulation, DBP is also synthesized in the liver. DBP binding prolongs the half-life of vitamin D and its metabolites and facilitates their uptake by target tissues [89]. Decreased levels of serum DBP were observed in patients who suffered from fulminant hepatic failure or chronic liver diseases. [90].

##### Kidney Disease

Within the proximal convoluted tubule cells of the kidney, the majority of circulating 1α,25(OH)_2_D is produced by the enzyme 1α-hydroxylase. Accordingly, renal pathology can be a key factor of vitamin D deficiency. Chronic kidney disease, defined by the presence of kidney damage or decreased kidney function for three or more months, affects approximately 20 million adults in the US [91]. Serum 1α,25(OH)_2_D level is positively associated with creatinine clearance in chronic kidney disease [92,93] and glomerular filtration deteriorates with disease progression [94]. Serum 1α,25(OH)_2_D levels are usually undetectable in end-stage renal disease. The activity of 1α-hydroxylase in renal glands is regulated by PTH and hypophosphatemia through PTH-induced enzyme synthesis and direct stimulation of enzymatic activity [89]. Impaired kidney function secondary to chronic kidney disease results in phosphate retention and later hyperphosphatemia, which is a potent inhibitor of renal 1α-hydroxylase activity. Loss of functioning kidney mass also causes decreased levels of this enzyme and a consequent deficiency in circulating 1α,25(OH)_2_D [95]. Moreover, low 25(OH)D levels can be observed in nephrotic-range proteinuria due to direct loss of DBP-bound 25(OH)D in the urine [94].

## 5. Impacts of Vitamin D on Musculoskeletal Health

### 5.1. Vitamin D and Bones

**Figure 2:**
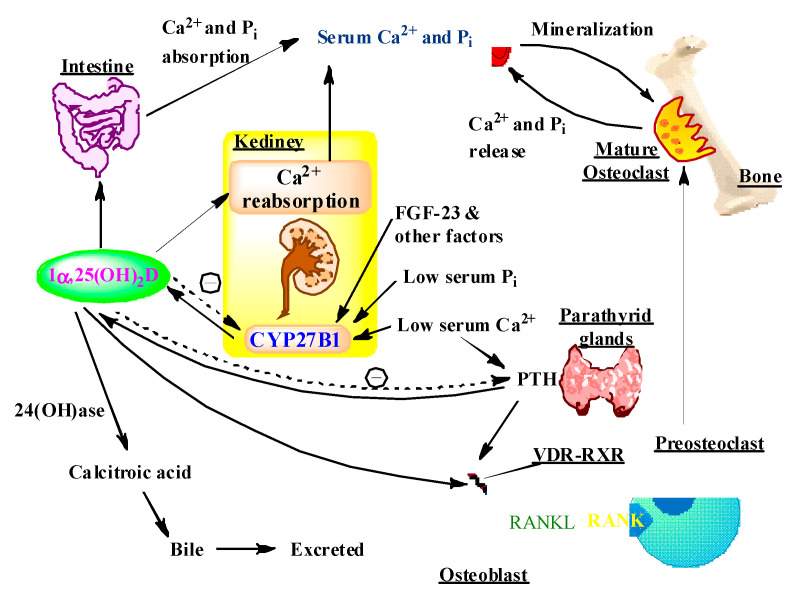
The effects of 1α,25(OH)_2_D on calcium and phosphorus homeostasis. 1α,25(OH)_2_D is produced by the kidney under the control of PTH by the parathyroid glands. PTH stimulates its production and 1α,25(OH)_2_D in turn inhibits the synthesis and secretion of PTH. 1α,25(OH)_2_D can also decrease its own synthesis through negative feedback. 1α,25(OH)_2_D enhances intestinal calcium and phosphorus absorption in the small intestine and calcium reabsorption in the kidney. 1α,25(OH)_2_D regulates bone formation and resorption by stimulation of preosteoblast proliferation and differentiation into osteoblasts. 1α,25(OH)_2_D also stimulates the expression of RANKL by osteoblasts, which stimulates the differentiation and subsequent activation of preosteoclasts into mature osteoclasts, the bone-forming cells which release calcium (Ca^2+^) and inorganic phosphorus (Pi) from the bone to maintain calcium and phosphorus levels in the blood. Adequate calcium and phosphorus levels promote the mineralization of the skeleton. 1α,25(OH)_2_D stimulates the expression of the renal 24-hydroxylase (24-OHase) to catabolize 1α,25(OH)_2_D to the water-soluble, biologically inactive calcitroic acid, which is excreted in the bile. Other factors, such as serum phosphorus, calcium, and fibroblast growth factor 23 can either increase or decrease the renal production of 1α,25(OH)_2_D.

The major role of vitamin D is to provide and maintain adequate calcium and phosphorus in the body to facilitate optimal metabolic function (Figure 2). Patients suffering from vitamin D deficiency absorb only 10–15% of dietary calcium and 50–60% of dietary phosphorus. When one has sufficient vitamin D, calcium and phosphorus absorption can increase 30–40% and 80%, respectively [5]. As the biologically active form, 1α,25(OH)_2_D accomplishes calcium homeostasis by interacting with VDR in the small intestinal cells, followed by complexing with retinoic acid X receptor (RXR) in the nucleus [96]. The resulting 1α,25(OH)_2_D–VDR–RXR complex together with co-regulatory proteins binds to the VDRE for the epithelial calcium channel [97]. The increased expression of the calcium channel permits more calcium to enter the cell, where the vitamin D-dependent calcium-binding protein calbindin-D_9K_ helps calcium’s translocation into the bloodstream. 1α,25(OH)_2_D also enhances calcium and phosphorus absorption in the small intestine and calcium reabsorption in the kidney [3]. The decreased serum-ionized calcium level is immediately recognized by the calcium sensor in the parathyroid glands, resulting in an increase in the expression, synthesis, and secretion of PTH [98,99,100]. PTH can decrease phosphorus reabsorption in the kidney, causing loss of phosphorus in the urine. The 1α,25(OH)_2_D-occupied VDR, together with PTH, enhances the expression of the plasma membrane protein receptor activator of nuclear factor-κB ligand (RANKL) on osteoblasts to increase the production of mature osteoclasts [98,101]. The mature osteoclasts mobilize calcium and phosphorus from the bone into the circulation via secretion of hydrochloric acid and collagenases. Thus, the major function of vitamin D is to maintain serum levels of calcium and phosphorus within the normal physiological range to support most metabolic functions, bone mineralization, and neuromuscular transmission [38,98].

### 5.2. Rickets

Among infants and young children, vitamin D deficiency is a common cause of bone deformities classically known as rickets [102]. Infants have a relatively high need of vitamin D because of their high rate of skeletal growth. In the first 4 months of life, an infant’s diet consists almost entirely of breastmilk and/or infant formula [103]. Despite the many benefits of breastmilk, the vitamin D content of breast milk is relatively low and ranges from 25 to 124 IU/L [104,105]. It has been hypothesized [106] that higher calcium or vitamin D supplementation to lactating mothers may result in increased calcium or vitamin D in the supply from the mother to the infant. However, neither calcium nor vitamin D intake affects the breastmilk calcium levels according to Basile et al. [107]. In other studies, it seemed that maternal vitamin D status had a great role in the milk vitamin D supply. Several RCTs have assessed the efficacy of the practice, using both regular and bolus dosing regimens (dose range 250–4000 IU/day or equivalent), and have observed it to raise both infant and maternal vitamin D status [96,106,108,109]. However, the follow-up period of these studies only extended to 7 months of age at the most [106]. Longer RCTs are required to assess the long-term benefit in future studies. In order to maintain safe vitamin D status, a daily supplement of 400 IU of vitamin D_3_ for breast-fed infants, as recommended by the Institute of Medicine of US, should be practiced [110].

### 5.3. Osteoporosis and Osteomalacia

Vitamin D deficiency also precipitates and exacerbates osteoporosis among adults and causes the painful bone disease called osteomalacia. Osteoporosis is a systemic skeletal disease characterized by decreased bone strength and increased risk of fractures. Although rickets is rare in the United States, osteoporosis affects one in three women and one in twelve men. Fractures of the wrist, hip, and vertebrae are the three main manifestations of osteoporosis and in at-risk populations cause excess mortality, a considerable economic burden, and a decrease in quality of life [111]. The risk of developing an osteoporotic fracture increases with advancing age. As a result, in the elderly, vitamin D levels have been suggested to be the best predictor of fracture risk [112]. In a study of more than 500 individuals with hip fractures, 95% were found to be vitamin D deficient [113]. A study of 82 patients with minimal trauma fracture found that all except two individuals had vitamin D levels less than 30 ng/mL [114]. Vitamin D trials have suggested that achievement of vitamin D sufficiency could reduce common osteoporotic fractures by 50 to 60% [115].

In addition to vitamin D deficiency, age, and gender, risk factors for osteoporotic fractures include Asian or Caucasian ethnic origin, low body weight, low dietary calcium intake, cigarette smoking, excessive alcohol consumption, long-term immobilization, low estrogen levels, glucocorticoid therapy, and low bone mineral density [116]. As serum 25(OH)D levels decrease with age, increased supplementation is necessary for most older individuals [117]. A randomized trial demonstrated that the probabilities of hip fractures and nonvertebral fractures among elderly women (78 to 90 years of age) who received 800 IU of vitamin D_3_ daily for 18 months were 43% and 32%, respectively, lower than among those who received placebo [118].

### 5.4. Muscle Weakness and Falls

Proximal muscle weakness is a prominent clinical feature of vitamin D deficiency. In addition, vitamin D deficiency is believed to be one of many factors contributing to the development of sarcopenia, the degenerative loss of muscle with aging, and may be an independent risk factor for postural sway and falls [119,120]. Falls resulting from neuromuscular dysfunction are the largest single cause of injury-related deaths in elderly people and lead to 40% of all nursing home admissions [121].

VDRs are located on the fast-twitch muscle fibers, which are the first to be recruited to prevent a fall [122]. It is theorized that 1α,25(OH)_2_D can bind to its receptor in muscle tissues, allowing protein synthesis and muscle cell growth, so that vitamin D can improve muscle strength and function, thereby preventing falls [123,124]. Furthermore, vitamin D may improve neuromuscular function and postural and dynamic balance, leading to a considerable increase in reaction time, and consequently, less falls and fractures [121,124]. A systematic review revealed that supplemental vitamin D given in daily doses of 800 to 1000 IU consistently had beneficial effects on muscle strength and balance [125]. Several RCTs have also reported positive effects of vitamin D supplementation on muscle function and fall prevention [126,127,128].

## 6. Impact of Vitamin D on Non-Musculoskeletal Health

**Figure 3:**
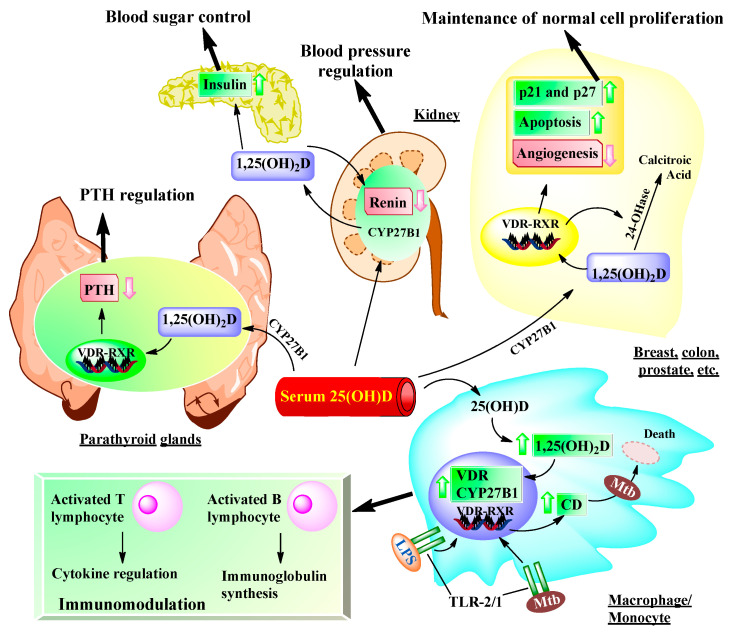
Metabolism of 25(OH)D to 1α,25(OH)_2_D for non-musculoskeletal functions. 1α,25(OH)_2_D not only regulates calcium and phosphorus homeostasis but can inhibit renin production in the kidney and stimulate the pancreas to secret insulin. 1α,25(OH)_2_D can also be converted from 25(OH)D through autocrine production and interacts with VDR in the breast, colon, prostate, and other tissues to regulate a wide variety of genes that control proliferation (such as enhancing expression of p21 and p27), inhibit angiogenesis, and induce differentiation and apoptosis. It is believed that the regulation of cell growth and maturation is important for decreasing risk of the cell becoming malignant. The upregulation of VDR and CYP27B1 expression occurs after the activation of toll-like receptor 2/1 (TLR2/1) in a macrophage or monocyte by an infectious agent such as *Mycobacterium tuberculosis (Mtb)* or its lipopolysaccharide. This results in an increase in the nuclear expression of cathelicidin, a cationic peptide capable of promoting innate immunity and the destruction of the infectious agents. The regulation of cytokine synthesis and immunoglobulin synthesis by activated T lymphocytes and activated B lymphocytes, respectively, is associated with the 1α,25(OH)_2_D, which is locally produced in monocytes and macrophages.

The existence of VDR in most tissues and cells in the body [129] results in a broad range of biological actions of 1α,25(OH)_2_D (Figure 3) in addition to regulating calcium and phosphorus homeostasis. The local production of 1α,25(OH)_2_D may be responsible for regulating up to 200 genes [130] that may facilitate many of the pleiotropic health benefits that have been reported for vitamin D [129,131].

### 6.1. Immunomodulatory Functions

**Figure 4:**
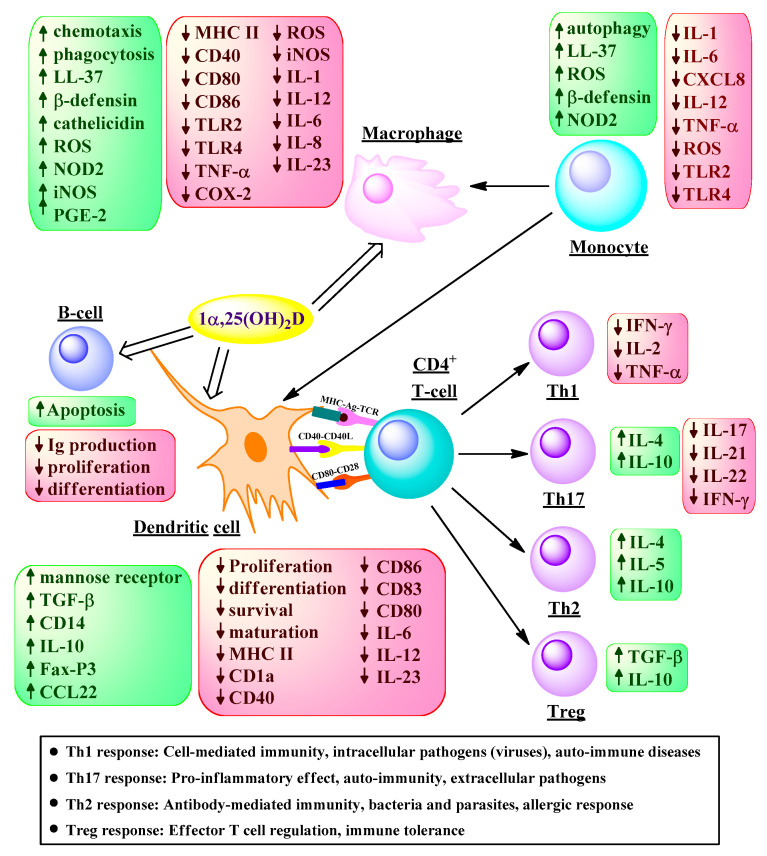
Mechanisms for adaptive immune responses to 1α,25(OH)_2_D. Monocytes produce more LL-37 and β-defensin, enhance autophagy and NOD2 (nucleotide‑binding oligomerization domain‑containing protein 2) expression, decrease the production of inflammatory cytokines and downregulate TLR2/4 expression. Differentiation into macrophages is increased; the chemotactic and phagocytotic responses of macrophages and the production of antimicrobial proteins such as cathelicidin are upregulated. However, the stimulatory capacity of the antigen-presenting cells (APCs) and T cells is decreased. At the level of the APC, 1α,25(OH)_2_D inhibits the differentiation into DCs, and thus stimulates effector CD4^+^ cells to differentiate into one of the four types of Th cells. Activated T cells also express VDR. 1α,25(OH)_2_D inhibits the development of Th1 cells associated with the cellular immune response, and promotes Th2 cells associated with humoral (antibody) mediated immunity, thereby indirectly promoting the T cell shift from a Th1 towards a Th2 phenotype. 1α,25(OH)_2_D also inhibits the development of Th17 cells, which play an essential role in combating certain pathogens and are linked to tissue damage and inflammation. Moreover, 1α,25(OH)_2_D favors Treg cell development via modulating DCs and by directly targeting T cells. Finally, B cells are also affected by 1α,25(OH)_2_D, demonstrating decreased immunoglobulin production, proliferation and differentiation, but increased apoptosis.

The immunomodulatory functions of vitamin D were first described more than 30 years ago [132,133,134]. 1α,25(OH)_2_D reduces the inflammatory response of T helper-1 (Th1) cells and suppresses antigen presentation by dendritic cells (DCs), both of which are involved in the autoimmune response [135]. Autoimmunity arises when Th1 cells are misdirected against self-proteins. The vitamin D metabolite 1α,25(OH)_2_D suppresses this pathology by regulating the differentiation and activity of CD4^+^ T cells, resulting in inhibition of the proliferation of Th1 cells [136]. 1α,25(OH)_2_D can also inhibit the differentiation of the DCs and their antigen-presenting ability, which stimulates T cells [137,138,139]; reduce the polarization of Th0 cells to Th1 cells [140]; and the secretion of Th1 cytokines, such as interleukin (IL)-2, IL-12 and interferon γ (IFNγ) [140,141,142]. Increased production of Th2 cytokines such as IL-4, IL-5, and IL-10 [143,144] has also been noted, which leads to a more balanced Th1/ Th2 response with less development of self-reactive T cells. Furthermore, 1α,25(OH)_2_D can reduce B cell proliferation and their differentiation into plasma cells [145], and increase the activity of the regulatory T cells (Tregs), which play an important role in maintaining immunological self-tolerance [136]. The immunomodulatory effects of 1α,25(OH)_2_D are summarized in Figure 4.

#### 6.1.1. Autoimmune Diseases

Autoimmune diseases are caused by dysfunctions of the body’s immune system leading to tissue damage. They are mediated by T and/or B cell activation in the absence of ongoing infection or other discernible causes [146]. The etiology and pathogenesis of most autoimmune disorders remain unclear and several factors have been implicated in their development. VDR was found in several cells and tissues in the immune system, such as lymphocytes, monocytes and DCs [147]. Numerous epidemiological studies, especially during the last decade, have reported an association between vitamin D deficiency and autoimmune diseases, including rheumatoid arthritis (RA), multiple sclerosis (MS), systemic lupus erythematosus (SLE), and inflammatory bowel disease (IBD) [136,148,149].

##### Rheumatoid Arthritis

RA is a chronic and systemic autoimmune disorder that primarily affects joints all around the body, including the wrists, hands, elbows, shoulders, knees, and ankles. RA may also develop into joint and tissue damage, resulting in severe disability and increased mortality [150]. Vitamin D’s effects on the innate immune system are predominantly through the TLRs and on the adaptive immune system through T cell differentiation, particularly the Th17 cell response. As Th17 cells are critical in the pathogenesis of RA, this has led to an interest in the effects of vitamin D deficiency in RA [151]. Epidemiological and observational evidence suggests a greater incidence of RA with increasing latitude, similar to the amplified risk of vitamin D deficiency [136,152]. For example, a higher prevalence of RA and lower 25(OH)D_3_ level (plasma 25(OH)D_3_ < 40 nmol/L) are noted in patients from northern Europe compared with those in southern Europe [153].

In an animal model study on the influence of the absence of vitamin D signaling in chronic arthritis, clinical symptoms of arthritis were aggravated in the VDR-deficient human tumor necrosis factor (TNF) transgenic mice. [154] Another study also found that the mice fed a diet supplemented with 1α,25(OH)_2_D had a 50% lower prevalence of collagen-induced arthritis compared to the control mice. [155] A recent meta-analysis and systematic review [156] analyzed 24 reports before May 2015 involving a total of 3489 patients. Overall, a negative association between serum vitamin D and RA activity was observed. Another systematic review and meta-analysis [157] identified five RCTs of vitamin D supplementation for at least 3 months (*n* = 640) and demonstrated that vitamin D supplementation could possibly reduce RA activity and its recurrence. However, inadequate randomization and allocation in some RCTs and relatively small sample numbers for the meta-analysis compromise the reliability of these data. In contrast, a cross-sectional study [158] performed on 100 RA patients (18 to 75 year-olds) with 25(OH)D tests within the prior 3 months found no statistically significant relationship between the 25(OH)D level and RA activity. Although vitamin D deficiency is more frequent among patients with RA [159], results published to date appear insufficient to fully elucidate the immunomodulatory role of vitamin D.

##### Inflammatory Bowel Disease

IBD is a group of chronic inflammatory conditions of the colon and small intestine, principally comprising CD and ulcerative colitis (UC). IBD is hypothesized to occur as a result of an abnormal immune response to enteric bacteria in genetically susceptible individuals [160]. A study on IL-10 knockout mice revealed that a low level of vitamin D was associated with accelerated bowel inflammation, while vitamin D_3_ supplementation ameliorated IBD symptoms in these mice [161,162]. In a double-blind RCT including 94 patients with CD in remission, daily oral treatment with 1200 IU of vitamin D_3_ increased the mean serum 25(OH)D_3_ concentration from 69 to 96 nmol/L and reduced the relapse rates over the 1-year follow-up (13% *vs*. 29%; *p* = 0.06) compared to the placebo group [163]. A systematic review and meta-analysis [164] identified 27 studies before February 2018 comprising 8316 IBD patients (3115 UC, 5201 CD) and provides evidence that 25(OH)D status is negatively correlated with the odds of IBD activity. Another systematic review and meta-analysis [165] including 18 RCTs (*n* = 908) from 1978 to 2018 concluded that vitamin D treatment in IBD patients can control the relapse rate of the disease. However, significant heterogeneity—vitamin dosage and treatment course; racial and regional diversity; age range and sex ratio—among the trials influence the judgments. Thus, more high-quality RCTs are still needed for evaluation.

##### Multiple Sclerosis

MS is an autoimmune disorder that affects the central nervous system. The body attacks its own insulating myelin sheaths covering the nerve cells in the brain and spinal cord, resulting in the partial impairment of the nervous system regarding communication, loss of motor and/or sensory function, lack of coordination, cognitive decline, and mental and psychiatric illness [166,167,168]. Although the exact etiology of MS is still unknown, oxidative stress resulting from reduced nicotinamide adenine dinucleotide phosphate oxidase (NOX) activation is thought to exacerbate the active demyelination and neurodegeneration in MS [169,170]. The increased reactive oxygen species (ROS) which are generated by the activation of NOX [171], will result in damage to the mitochondria to generate further ROS [170,172]. This buildup of ROS can reduce the efficiency of glutamate transporters, leading to raised glutamate concentrations and consequently enhancement in excitotoxic damage [173]. Additionally, the inevitable increase in Ca^2+^ will activate the permeability transition pores, resulting in apoptosis [172].

In mesencephalic dopaminergic neurons, vitamin D can alleviate ROS-induced neurotoxicity by increasing the level of glutathione [174]. There is increasing genetic evidence to suggest that vitamin D deficiency is an important risk factor in MS [175,176,177,178]. One study showed that vitamin D regulates > 80% of MS-related genes. A probable reason that lower levels of 1α,25(OH)_2_D resulting from CYP27B1 mutations can give rise to disruptions to the critical gene–environment interactions important for the developing immune or nervous system, which then predispose one to MS, has been suggested [179]. In brief, genetic polymorphisms within key enzymes involved in vitamin D metabolism, and within the VDR, have been associated with the risk of developing MS [180].

Low serum 25(OH)D levels (~20 ng/mL) are usually observed in MS patients as early as the beginning of the disease [181]. In a recent prospective cohort study (170 natalizumab-treated patients with relapsing-remitting MS) [182], blood samples were collected during winter 2009–2010 (baseline) with follow-up during the subsequent winter. It was found that recommending oral vitamin D supplements in patients with vitamin D insufficiency was associated with a significant increase in serum 25(OH)D level (95% CI, −0.026 to −0.003) and decreases in the annualized relapse rate (*p* = 0.02). In contrast, a retrospective study [183] including 554 MS patients over three years of follow-up only observed a significant correlation between 25(OH)D status and the subsequent relapse risk in younger MS patients (≤37.5 years; OR = 0.872, per 10 nmol/L 25(OH)D, *p* = 0.041), while no relationship was found between serum 25(OH)D level and disability or disability progression. As heat aggravating the MS symptoms results in fewer outdoor activities and consequently less exposure to UVB in disabled patients, the reverse causality makes it more difficult to interpret the results. However, vitamin D supplementation in moderate doses for MS patients is recommended for essentially correcting their vitamin insufficiency.

##### Asthma

Asthma is a common chronic inflammatory disease of the airways of the lungs. It is characterized by bronchospasm; reversible airflow obstruction; and variable and recurring symptoms, including episodes of wheezing, coughing, chest tightness, and shortness of breath [184]. Although the role of vitamin D in asthma is not currently well understood, some putative correlations between vitamin D and asthma have been reported. Significant associations between polymorphisms in the VDR gene with asthma have been reported in several genetic association studies, but this has not been consistently replicated [185,186,187]. It was also observed that children who took vitamin D daily had a relative risk reduction of 93% for having asthma attacks compared with children who did not take vitamin D supplements [188]. A community-based prospective study on the transition of children to an allergic asthma phenotype showed that 25(OH)D_3_ levels at ages 6 and 14 were negatively associated with concurrent allergic phenotypes, particularly in boys. Vitamin D levels at age 6 were also significant predictors of subsequent atopy/asthma-associated phenotypes at 14 years of age [189]. Potential mechanisms of the action of vitamin D might include promoting lung immunity, decreasing inflammation, slowing cell cycling, reducing hyperplasia, and enhancing the effects of exogenous steroids [190].

However, a number of pieces of evidence were not consistently supportive of a causal role for vitamin D in reducing the risk of asthma. In a nested case-control study, no association between baseline serum 25(OH)D concentration < 50 nmol/L and asthma in men (OR = 1.47; 95% CI, 0.93–2.32) or women (OR = 0.94; 95% CI, 0.67–1.32) was noted [191]. Data from a study on the prospective associations of 25(OH)D_2_ and 25(OH)D_3_ with asthma, wheezing, flexural dermatitis, and lung function in children showed interesting results. It is likely that higher serum 25(OH)D_2_ concentration was associated with reduced risks of wheezing and flexural dermatitis and better lung function, while serum 25(OH)D_3_ concentration was positively associated with wheezing and flexural dermatitis. No correlation was found between 25(OH)D_3_ or total 25(OH)D concentration and diagnosed asthma or lung function [192]. A systematic review of observational studies assessing relationships between maternal vitamin D intake during pregnancy (*n* = 4), maternal serum 25(OH)D concentration in pregnancy (*n* = 2) or cord blood 25(OH)D concentration (*n* = 2) and asthma, reported conflicting results that make it difficult to establish any clear relationship between maternal serum 25(OH)D concentration and the development of asthma in offspring [193].

##### Type 1 Diabetes (T1D)

T1D, an autoimmune disorder which induces loss of insulin-producing β-cells in the pancreas, is usually diagnosed in children and younger adults. It has been postulated that immune-modulatory actions of vitamin D decrease the cytokine production and lymphocyte proliferation, thereby preventing the destruction of β cells and subsequent development of T1D [194,195,196]. An association has been noted between VDR polymorphism and T1D in several genetic studies [197,198,199]. Polymorphism in the *CYP27B1* gene was found to reduce the level of active 1α-hydroxylase, and subsequently suppress the conversion of 25(OH)D to 1α,25(OH)_2_D, resulting in an increased predisposition to T1D [200]. There is also a large body of evidence linking a lack of vitamin D early in life to the development of T1D. In vivo studies have shown that vitamin D deficiency leads to decreased insulin production from pancreatic β cells, leading to impaired glucose tolerance [201]. Vitamin D supplementation during infancy was reported to confer partial protection against β-cell autoimmunity [202]. The risk of islet cell antibodies in offspring was decreased by 63% with a single standard deviation (156 IU) increase in recalled maternal dietary vitamin D intake during pregnancy [203]. Similarly, higher intake of maternal cod liver oil, a source of vitamin D, was associated with a decreased risk of type 1 diabetes in offspring during pregnancy [204].

##### Systemic Lupus Erythematosus

SLE is a common autoimmune disease in which the body’s immune system produces antibodies against its own healthy tissues. These autoantibodies specific for DNA, RNA or proteins bind to nucleic/amino acids to form immune complexes, which contribute to the damage of small blood vessels, especially in the kidneys. Patients with SLE generally have abnormal B and T cell function, along with rashes, arthritis, kidney disease and central-nervous-system involvement [203].

Higher severity of SLE has been shown to be associated with lower levels of vitamin D [205]. However, whether lower vitamin D levels cause disease or are a consequence of the disease or its treatment is not clear. Vitamin D deficiency could be due to many factors in these patients, such as frequent use of photoprotection, since the patients are usually photosensitive, chronic steroid use causing a variation in vitamin D metabolism, renal involvement from SLE resulting in decreased hydroxylation of 25(OH)D or the formation of anti-vitamin D antibodies in a subset of patients with SLE [205].

In murine models of experimental SLE, administration of 1α,25(OH)_2_VD_3_ or an analog prevented proteinuria and increased the life spans of mice [135,206,207]. An in vitro experiment on the treatment of peripheral blood mononuclear cells isolated from 25 SLE patients with 50 nM of 1α,25(OH)_2_VD_3_ showed that vitamin D has regulatory effects on cell cycle progression, apoptosis and apoptosis-related molecules in lupus patients [208]. In addition to its potential benefit to SLE patients, vitamin D is known to present an immune-inflammatory-modulatory effect that can benefit musculoskeletal and cardiovascular manifestations of SLE [209]. The results from one investigation demonstrate that vitamin D can positively modify endothelial repair mechanisms and thus endothelial function in SLE patients that are susceptible to CVDs [210]. Other than vitamin D levels, VDR gene polymorphisms may also play a role in the risk of SLE. A recent meta-analysis concluded that BsmI B may be a risk factor for SLE onset for the overall population and that the FokI FF genotype is a risk factor in Asians for SLE susceptibility [211]. This was further confirmed by a recent follow-up study, which found a positive association between VDR polymorphisms and SLE severity, especially for the FokI CT and TaqI TT genotypes in 170 SLE patients [212]. Conversely, it has been shown that increasing levels of vitamin D intake were not associated with decreased risk of developing SLE in the Nurses’ Health Study for up to 22 years of follow-up of 186.389 women [213].

#### 6.1.2. Innate Immunity and Infectious Diseases

In the days when rickets was rampant, children with this disorder were at higher risk of death from respiratory infections [46,214]. Vitamin D in its autocrine role has been recognized for more than 20 years as playing a role in modulating the innate immune response, and its deficiency is related to plenty of infectious diseases, such as tuberculosis, pneumonia, influenza, septicemia and periodontal disease [214,215,216]. This possible role has been suggested by the presence of VDRs and CYP27B1 in various cells of the immune system, including B and T lymphocytes, macrophages and DCs. Upregulation of the expression of both CYP27B1 and VDR, involved in the initiation of antimicrobial responses, requires adequate levels of vitamin D [217,218]. When the cells of the immune system such as macrophages ingest an infectious agent, such as tuberculosis bacillus, the TLRs are activated, resulting in signal transduction to increase the expression of VDR and CYP27B1. In turn, CYP27B1 gene enhances the local conversion of 25(OH)D to 1α,25(OH)_2_D, which subsequently acts in an autocrine/paracrine manner through VDR. This increases the formation of antimicrobial peptides such as cathelicidin and defensin beta 4 that facilitate the killing of mycobacteria [218]. Other roles vitamin D plays in the maturation of macrophages include the production of macrophage-specific surface antigens and the secretion of the lysosomal enzyme acid phosphatase and hydrogen peroxide [147]. Together with the enhancement of the transcription of endogenous antibiotics such as cathelicidin and defensins, these explain why vitamin D possesses antimicrobial activity despite it inhibiting immune reactions in general.

Cell studies have proposed that the infected macrophage is unable to produce sufficient 1α,25(OH)_2_D to upregulate production of cathelicidin in cases of vitamin D deficiency, and higher 1α,25(OH)_2_D levels enhanced the bactericidal activity of human macrophages against *Mtb* [219]. In a murine model of heart allograft, 1α,25(OH)_2_D and one of its analogs prolonged allograft survival, suggesting that vitamin D might be used as adjuvant therapy in association with immunomodulating drugs in organ transplantation [220]. Clinically, it has been noted in RCTs that vitamin D co-therapy substantially improved the response to standard antitubercular therapy in patients with advanced pulmonary tuberculosis [221], and as a secondary outcome, reduced risk for influenza in postmenopausal black women who received vitamin D [222]. Additionally, the phagocytic function of human macrophages was enhanced in individuals who received vitamin D supplementation (a single oral dose of 2.5 mg) compared to those who took a lactose placebo [223].

##### Vitamin D and COVID-19

The world is in the grip of the COVID-19 pandemic, which has had over 100 million confirmed cases and over 2 million deaths in 216 countries worldwide (as of February 10, 2021; World Health Organization). The leading cause of death is acute respiratory distress syndrome (ARDS), since the lungs are a major target for COIVD-19 virus. ARDS is mainly caused by a “cytokine storm,” a hyperactive immune response triggered by the infection and further magnified by attendant oxidative stress. The immune cells release all types of cytokines, such as IFNs, ILs, chemokines, colony-stimulating factors and TNF, leading to hyperinflammation, lung damage and mortality. In severe cases of COVID-19, other organs and systems are also damaged [224].

Recently, vitamin D deficiency has emerged as a potential risk factor predisposing one to COVID-19 [224,225]. Pulmonary alveolar macrophages are induced to express CYP27B1 and the vitamin D receptor by pathogens [226]. 1,25(OH)_2_D can regulate the innate immune response through a number of mechanisms, including suppression of IL-6 production by innate monocytes [227], which have recently been identified as being involved in the atypical innate immune responses induced by COVID-19 [228,229]. 1,25(OH)_2_D also downregulates TLRs and directly inhibits TNF/NFκB and IFNγ signaling pathways. As for adaptive immunity, 1,25(OH)_2_D limits the DC maturation and the ability of DCs to present antigen to T cells, and shifts the T cell profile from the proinflammatory Th1 and Th17 subsets to Th2 and Treg subsets, leading to proinflammatory inhibition [226]. The administration of vitamin D in a study showed that it could reduce the expression of renin, angiotensin II and angiotensin 1 receptors and increase angiotensin-converting enzyme 2 and angiotensin activities, resulting in reduction of the inflammatory process and lung damage [230]. Albeit most results come from studies with a variety of pathogens, viral and bacterial, the effect of vitamin D on regulating COVID-19 lung immunopathology needs to be rigorously evaluated in the future. A systematic meta-analysis [231] of 25 RCTs found that vitamin D protected against acute respiratory tract infection. Several other systematic reviews and meta-analyses [232,233,234,235] observed a potentially positive association between vitamin D deficiency and COVID-19 infection, severity and mortality. However, additional evidence with larger populations and prospective study designs in RCTs are needed for further evaluation.

### 6.2. Cancers

Observations have demonstrated increases in risks of developing several cancers and cancer mortality with increasing latitude, and longer survival for patients diagnosed with certain malignancies during summer months than winter months [236,237]. Since the intensity of sun exposure decreases with increasing latitude, and on the basis that sun exposure is a proxy for vitamin D status, it was first suggested that vitamin D might influence cancer by Garland and Garland [238]. They proposed that the high rate of colon cancer seen in the Northern US compared with the Southern US was due to the UV light-induced production of vitamin D in the skin. Later, the hypothesis was extended to 18 different types of cancer [239]. This is also evidenced by some cell culture studies and animal studies. It was observed that 1α,25(OH)_2_D_3_ inhibited growth of malignant cell culture lines [240,241] and that reductions in tumor development and growth occurred in animals injected with 1α,25(OH)_2_D_3_ analogs [242,243,244,245,246]. In addition, some other animal studies have shown that severe vitamin D deficiency [243,247,248], or deletion of the VDR gene [249,250,251], increases cancer risk.

The proposed mechanisms of vitamin D’s anticancer effects via transcriptional regulation mainly encompass: (1) anti-proliferation, (2) induction of apoptosis, (3) stimulation of differentiation, (4) reduced inflammation, (5) inhibition of invasion and metastasis, and (6) inhibition of angiogenesis [252]. Anti-tumor studies of 1α,25(OH)_2_D_3_ and vitamin D analogs in different types of cancer cell lines are classified by each of these effects and summarized in the Supporting Information (Table S1).

Some population-based studies show that low serum 25(OH)D levels are associated with increased risks of cancers of the colon [253,254,255,256,257], breast [258,259], and prostate [260,261], and other cancers [262,263,264]. However, together with cell culture and animal studies, although a growing consensus insists that vitamin D is closely related to cancer risk, some population-based studies showed inconsistent associations. One cohort study followed white adults (*n* = 1621; mean age, 74 years) in the US for a median of 11 years and reported no association between seasonally adjusted serum 25(OH)D concentration and cancer [265]. Vitamin D’s effects on cancer were also evaluated in the “VITamin D and OmegA-3 TriaL (VITAL),” an RCT in 25,871 older participants in the United States who were randomized to 2000 IU of vitamin D daily, 1 g of omega-3 daily or placebo [266]. After a median follow-up time of 5.3 years, it was concluded that supplementation with vitamin D did not result in a lower incidence of invasive cancer than placebo. Interestingly, an inverse relationship between 25(OH)D level and cancer risk was also observed. A meta-analysis reported that higher serum 25(OH)D concentration was associated with significant increases in risk in basal cell skin cancer and non-melanoma skin cancer [267]. Stolzenberg-Solomon and co-authors reported a significant increase in pancreatic cancer risk associated with higher (≥100 nmol/L) compared to lower (<25 nmol/L) serum 25(OH)D concentration [268].

The studies are, however, subject to confounding by behavioral and lifestyle factors that influence serum 25(OH)D concentrations, and the conflicting results made it difficult to conclude any relationship between vitamin D and cancer risk. In the large control population of the “Cohort consortium vitamin D pooling project of rarer cancers,” including men and women from US, Chinese and Finnish cohorts, several correlates of serum 25(OH)D concentration were measured [269]. Statistically significant positive correlates of serum 25(OH)D concentration included male sex, vigorous physical activity, and alcohol intake. Significant inverse correlates were BMI, diabetes, sedentary behavior, and smoking. Data from two studies showed that gender and ethnic background seem to affect this association too. One study followed adults in Germany (*n* = 9580; age, 50–74 years) for more than 8 years and reported an association between low serum 25(OH)D concentration (season-specific ranges, 30–36 nmol/L) and increased risk for any cancer in men but not in women [270]. Another study of five ethnic groups in the US (white, African American, Native Hawaiian, Japanese, and Latino) reported an inverse association between breast cancer risk and 25 nmol/L increases in plasma 25(OH)D concentration (OR = 0.66; 95% CI, 0.48–0.90) in whites but not in other ethnic groups [259].

### 6.3. Cardiovascular Diseases

CVDs include a range of diseases of the heart and blood vessels, such as coronary artery diseases, congenital heart disease, stroke, hypertension, and vascular dementia [271]. Smoking, high blood pressure, high blood cholesterol, excessive alcohol consumption, physical inactivity, obesity, diabetes, family history, sex, and age are common risk factors for CVDs [271]. CVDs are predominantly caused by atherosclerotic deposits in large and medium-sized arteries. Besides lipid deposition and inflammation, calcification is also a significant component of advanced atherosclerotic lesions. Therefore, the impact of calcium intake in relation to atherosclerotic CVD should be taken into consideration. Since vitamin D has the potential to increase calcium absorption in the presence of high calcium intakes, it is also biologically plausible that vitamin D might increase vascular calcification, and as a consequence, increase CVD risk. Indeed, available data demonstrate that vitamin D exerts a biphasic dose–response curve (a “U” curve) on vascular calcification with the negative consequences of not only vitamin D excess but also of vitamin D deficiency [272]. However, both lower and upper boundaries of the optimal range for 25(OH)D levels for cardiovascular health remain controversial.

VDRs now are known to exist in heart and vascular tissue where they regulate the expression of multiple genes. Vitamin D deficiency has been found in multiple populations with CVD risk factors, and another study suggested that vitamin D deficiency is a risk factor itself for developing CVDs [130,273]. Several lines of evidence have been suggested in support of a biologically plausible relationship between serum 25(OH)D concentration and cardiovascular events. A murine model has shown that VDR knockout mice develop heart failure despite normalized calcium concentrations [274]. Some other animal studies have also suggested a link between ingested vitamin D and atherosclerosis [275,276].

In a prospective study, a decreased risk of coronary heart disease (CHD) was found to associate with serum 25(OH)D concentrations (>47.7 nmol/L), significantly in women [277]. In addition, Robinson-Cohen and coauthors reported that lower 25(OH)D concentrations were associated with increased CHD risk in white and Chinese participants but not in black and Hispanic participants [278]. Conversely, a few prospective cohort studies reported no association between serum 25(OH)D concentration and CVD incidence [266,279,280].

A leading explanation for a relationship between vitamin D deficiency and CVD is that chronic vitamin D deficiency leads to secondary hyperparathyroidism, which then can act through at least three pathogenic pathways to increase CVD risk: (1) increased insulin resistance and pancreatic β-cell dysfunction, predisposing one to metabolic syndrome and diabetes; (2) activation of the renin-angiotensin system, increasing blood pressure and leading to left ventricular hypertrophy (with subsequent myocyte apoptosis and cardiac fibrosis); (3) stimulation of systemic and vascular inflammation, augmenting atherogenesis [281,282]. In hemodialysis patients with secondary hyperparathyroidism, intravenous treatment with 1α,25(OH)_2_D was found to significantly reduce myocardial hypertrophy [283]. This might be due to the effect of 1α,25(OH)_2_D on the myocardium or renin-angiotensin system, or the direct effect of PTH on the heart muscle.

However, data from some studies show that PTH elevation appears to explain only part of the association of CVD risk with vitamin D deficiency. [284,285]. Some other non-PTH-related CVD mechanisms have proposed that vitamin D can affect blood pressure through the reticular activating system, vascular calcification, smooth muscle cell proliferation, and inflammation [286]. Additional recent evidence suggests that vitamin D supplementation may reduce endothelial dysfunction and arterial stiffness, and thus improve vascular function [287]. Further, it has been reported that high 25(OH)D levels can protect against cardiovascular risk by promoting the formation of large high-density lipoprotein particles, affecting reverse cholesterol transport [288]. Other molecular mechanisms of CVD likely await discovery.

### 6.4. Neuropsychological Functioning

Neuropsychological functioning, encompassing cognitive function, depression, dementia, autism, and schizophrenia, involves specific psychological processes and behaviors regulated by brain function. The effect of vitamin D on brain function is an area of growing interest, as the role of vitamin D in brain function is becoming clearer. [289,290]. From a biological perspective, vitamin D receptors and 1α-hydroxylase have been identified in the cerebral cortex and cerebellum. This suggests that the brain has the ability to synthesize 1α,25(OH)_2_D within many cell types and regions, predominantly in the hypothalamus and the large neurons within the substantia nigra [291]. Functionally, vitamin D contributes to neuroprotection by modulating the production of nerve growth factor [291], neurotrophin [292], glial cell-derived neurotrophic factor [293], nitric oxide synthase [294], and choline acetyltransferase [295]. This provides the possibility that vitamin D might impact various aspects of brain function (such as mood and cognition) and diseases caused by abnormal brain function (such as autism and schizophrenia).

Animals deprived of vitamin D early in development show evidence of abnormal brain development [296]. Data from those studies have shown that after administration of selective dopamine toxins, such as 6-hydroxydopamine, pretreating animals with 1α,25(OH)_2_D for one week still preserves dopaminergic function, indicating vitamin D may also have a neuroprotective effect on dopaminergic pathways in the brain [297,298]. A systematic review of observational studies including 25 cross-sectional (*n* = 48,680) and six prospective studies (*n* = 10,896) [299] assessed the association between serum 25(OH)D concentration and cognitive function; 18 out of 25 cross-sectional studies reported a significant decline in one or more cognitive function test or a higher frequency of dementia with lower vitamin D levels or intake. Four out of six prospective studies showed a higher risk of a worse outcome after a follow-up period of 4–7 years in participants with lower vitamin D levels at baseline. Other studies failed to show an association.

### 6.5. UV-Induced Damage

As indicated above, humans once depended on sunlight for their vitamin D requirements. Although cutaneous vitamin D_3_ synthesis is a major benefit from UV irradiation, other vitamin D-independent positive effects exist, such as reducing blood pressure by induction of nitric oxide, and elevating energy and mood through the release of endorphins [300]. However, UV is also known to be responsible for approximately 65% of melanoma cases, and 90% of non-melanoma skin cancers [301]. It is well established that UV radiation causes promutagenic DNA lesions in the skin directly by creation of genotoxic free radicals. These radicals disrupt double bonds in adjacent pyrimidines and thus distort the DNA helix [302]. The UV-induced DNA damage also occurs indirectly by generation of ROS and reactive nitrogen species (RNS). These species are known to cause oxidative and/or nitrosative damage to DNA, leading to alteration of the coding sequence, and thus tumor growth and progression [303]. Overexposure to UV radiation leads to photoimmune suppression [304,305], which depresses cell-mediated immune reactions that would normally destroy developing skin tumors [306,307]. Photoaging of the skin via wrinkles and dyspigmentation, followed by the formation of benign or malignant tumors, is also a consequence caused by UV irradiation [308].

The photoprotective effect of vitamin D compounds against UV-induced photoproducts such as thymine dimers in solar irradiated skin has been reported by various groups [309,310,311]. Reduction of two other major UV-induced photolesions, 8-oxo-7,8-dihydro-2’-deoxyguanosine and 8-nitroguanine by 1,25(OH)_2_D_3_ in irradiated human ex vivo skin explants was also demonstrated [310]. 1,25(OH)_2_D_3_ has been shown to enhance DNA repair, reduce inflammation and improve cell survival by reducing RNS such as nitrite and 3-nitrotyrosine and augment p53 expression [312]. 1,25(OH)_2_D_3_ was also found to have a regulatory effect on the transcription factor AP-l and MMPs via the VDR and attenuated TNF-α induced MMP3, thereby probably reducing UV-induced collagen degradation in skin [313].

## 7. Vitamin D Toxicity

Vitamin D toxicity can lead to hypercalcemia and hypercalciuria caused by increased intestinal calcium absorption and mobilization of calcium from the bone. These toxic effects include calcification of soft tissue, diffuse demineralization of bones, and irreversible renal and cardiovascular disorders [314,315]. The mechanism of how vitamin D toxicity might arise is presently unclear. Proposed mechanisms are based on increased concentrations of the active metabolite of vitamin D reaching the VDR in the nuclei of target cells and causing gene overexpression. Three main hypotheses have been proposed [316]: increased plasma 1α,25(OH)_2_D concentrations lead to increased cellular concentrations of 1α,25(OH)_2_D; serum 25(OH)D levels exceed DBP binding capacity and free 25(OH)D enters the cells and has direct effects on gene expression; concentrations of a number of vitamin D metabolites, especially vitamin D itself and 25(OH)D, exceed the DBP binding capacity, causing release of free 1α,25(OH)_2_D, which enters target cells.

In fact, prolonged sunlight exposure does not lead to excess production of cutaneous vitamin D because endogenously produced previtamin D_3_ and vitamin D_3_ are destroyed by the sun [317]. The only cause of toxicity is due to nonintentional or intentional ingestion of excessively high quantities of vitamin D for a prolonged period of time [318,319]. Cases of vitamin D toxicity resulting from ingestion of over-fortified milk have also been reported [320,321]. However, indeed, there is a comfortable safety margin between vitamin D toxicity and the intakes required for optimal vitamin D status. A risk assessment for vitamin D reviewing the totality of the toxicity data concluded that there were no cases of intoxication reported for daily intakes of <30,000 IU for extended periods or at serum 25(OH)D levels < 200 ng/mL (500 nmol/L) [315].

## 8. Conclusions

Vitamin D has extensive and exciting potential and its potential benefits are under exploration. Despite the remarkable progress made recently, the available evidence on the relationship between vitamin D and health is far from complete. One limiting factor of the studies is that the existing 25(OH)D assays are excessively variable, and the lack of a standard reference material exacerbates this problem. The findings from these studies should be interpreted with caution, since several behavioral and lifestyle factors, such as smoking and diet, can influence the serum 25(OH)D concentrations as well. Indeed, only a few studies took confounders including baseline vitamin D status, BMI, age, pubertal stage, season, sickness, compliance and physical activity into consideration. These biases can be neglected by randomly assigning thousands of participants to receive or not receive the treatment. Biological flaws, referring to limitations in the design of primary studies that prevent the evaluation, also constitute a possible reason that meta-analyses of vitamin D have failed to demonstrate efficacy [322]. Due to the limitations in the evidence concerning vitamin D, further genomic investigations and functional studies in larger groups need to be performed to confirm previous findings. Furthermore, not only the optimal serum 25(OH)D level for the definition of vitamin D deficiency but also the relevant functional outcomes for bone and other health aspects need determination and validation so as to assess vitamin D status across the life cycle.

## Supplementary Materials

The following are available online at https://www.mdpi.com/1422-0067/22/4/2128/s1.

## Abbreviations

1α,25(OH)_2_D	1α,25-dihydroxyvitamin D
25(OH)D	25-hydroxyvitamin D
APC	antigen-presenting cell
ARDS	acute respiratory distress syndrome
BMI	body mass index
CYP	cytochrome P450
CYP27B1	cytochrome P450 27B1
CD	Crohn’s disease
CHD	coronary heart disease
CVD	cardiovascular disease
DC	dendritic cell
DBP	vitamin D binding protein
DU	Dobson unit
HPLC	high performance liquid chromatography
IBD	inflammatory bowel disease
IL	interleukin
IFN	interferon
IU	international unit
*Mtb*	*Mycobacterium tuberculosis*
MS	multiple sclerosis
NOX	reduced nicotinamide adenine dinucleotide phosphate oxidase
PBMC	peripheral blood mononuclear cell
PTH	parathyroid hormone
RA	rheumatoid arthritis
RANKL	receptor activator of nuclear factor-κ B ligand
RCT	randomized controlled trial
RXR	retinoid-X receptor
ROS	reactive oxygen species
RNS	reactive nitrogen species
SLE	systemic lupus erythematosus
SZA	solar zenith angle
Th1	T helper 1
TLR	toll-like receptor
TNF	tumor necrosis factor
Treg	T regulatory cell
T1D	type 1 diabetes
UVB	ultraviolet B
UC	ulcerative colitis
VDR	vitamin D receptor
VDRE	vitamin D response element
