# Supplementary material for: Non-Musculoskeletal Benefits of Vitamin D beyond the Musculoskeletal System
Source: Int J Mol Sci. 2021 Feb 21;22(4):2128. doi: 10.3390/ijms22042128 (PMC7924658; doi:10.3390/ijms22042128)
Supplement: Supplementary file 1 [file ijms-22-02128-s001.pdf]

Non-musculoskeletal benefits of vitamin D beyond the musculoskeletal system

Table S1. Anti-tumor effects of 1α,25$(OH)_2D_3$ and vitamin D analogs on different cancer cell lines

Cell lines	Treatment	Key Effects on cancer cell lines
**1. Anti-proliferative effects**		
(Breast cancer)		
MCF-7	1α,25$(OH)_2D_3$	Suppress expression of the proto-oncogene *c*-MYC and increase the level of its antagonist, the transcriptional repressor MAD1/MXD1 [1]
	1α,25$(OH)_2D_3$	Inhibition of cyclin D1/CDK4, the E2F transcription factors and thus repression of cyclin A protein; induction of CDK inhibitor p21 and block cyclin A(E)/CDK 2 complexes; down-modulation of the *c*-MYC oncoprotein [2]
	1α,25$(OH)_2D_3$	Induction of expression of BRCA1 mRNA and protein as well as transcriptional activation from the BRCA1-promoter [3]
	1α,25$(OH)_2D_3$	Inhibition of the MAPK cascade through suppression of Src tyrosine kinase and stimulation of PTP (a non-genomic mechanism) [4]
T-47D, MCF-7	1α,25$(OH)_2D_3$	Reduction of EGF receptor levels [5]
(Prostate cancer)		
LNCaP	1α,25$(OH)_2D_3$	Induction of the formation of VDR/Sp1 complex and inhibition of $p45^{Skp2}$ transcription through a Sp1- and HDAC1-dependent pathway [6]
	1α,25$(OH)_2D_3$	Reduction of nuclear Cdk2 for cyclin binding and activation; impairment of cyclin E-Cdk2-dependent p27 degradation through cytoplasmic mislocalization of Cdk2 [7]
	1α,25$(OH)_2D_3$ + genistein	Upregulation of VDR protein by increasing VDR stability and up-regulation $p21^{waf1}$ levels [8]
	1α,25$(OH)_2D_3$ + genistein	Reduction of PGE2 levels through COX-2 suppression and 15-PGDH induction; inhibition of PGE2 signaling through reduction in EP receptor levels [9]
	1α,25$(OH)_2D_3$	Downregulation of FAS mRNA due to feedback inhibition of FAS expression by long chain fatty acyl-CoAs [10]
LNCaP, PC3	1α,25$(OH)_2D_3$ + 9-*cis*-RA	Inhibition of *hTERT* gene transcript and telomerase activity [11]
	1α,25$(OH)_2D_3$	Induction of *BRCA1* gene expression via transcriptional activation by factors induced by the VDR [3]
	1α,25$(OH)_2D_3$	Inhibition of prostaglandin metabolism through downregulation of COX-2 mRNA and upregulation of 15-PGDH mRNA expression and reduction of PG receptor mRNA levels [12]

LNCaP, PC3, DU145	19-nor-hexafluoride; vitamin D3	Upregulated protein expression of $p21^{waf1}$ and $p27^{kip1}$ [13]
ALVA-31, LNCaP	$1\alpha,25(OH)_2D_3$	Induction of p21 mRNA and protein levels [14]
PC3	$1\alpha,25(OH)_2D_3$ + TSA	Upregulated GADD45α and suppression of responsiveness of VDR [15]
C4-2	$1\alpha,25(OH)_2D_3$	Downregulation of c-Myc and Bcl-2 expression independently of Rb protein, leading to a decrease in E2F and its target genes [16]
LNCaP, CWR22R	$1\alpha,25(OH)_2D_3$	Inhibition of CDK2 activity and induction of G0/G1 arrest through androgen/AR signaling [17]
(Colorectal cancer)		
CaCo-2	$1\alpha,25(OH)_2D_3$	Inhibition of expression of EGFR mRNA and protein [18]
	$1\alpha,25(OH)_2D_3$ + TGF-β	Activation of TGF-β and thus upregulation of IGF-IIR expression [19]
	$1\alpha,25(OH)_2D_3$ + butyrate	Upregulation of VDR, followed by a stimulation of the negative cell cycle regulator $p21^{Waf1/Cip1}$ expression and by a downregulation of cdk 6 and cyclin A [20]
SW480-ADH	$1\alpha,25(OH)_2D_3$	Downregulation the expression of *ID2* genes [21]
HT-29	$1\alpha,25(OH)_2D_3$; EB1089; CB1093	Inhibition of IGF-II signalling by inhibiting IGF-II secretion and increasing the production of IGF-binding protein-6 [22]
LS180	$1\alpha,25(OH)_2D_3$	Repression of c-FOS and c-MYC genes expression via a direct action of both VDR and the TCF4/β-catenin regulatory complex [23]
(Ovarian cancer)		
OVCAR3	$1\alpha,25(OH)_2D_3$	Increased $p27^{Kip1}$ protein expression and stability by downregulation of Cyclin E/CDK2 activity and reduction of the abundance of Skp1-Cullin-F-box protein/Skp2 ubiquitin ligase [24]
	$1\alpha,25(OH)_2D_3$	Cause of cell cycle arrest at the G2/M transition through p53-independent induction of GADD45 [25]
(Pancreatic cancer)		
BxPC-3; Hs 700T SUP-1	$1\alpha,25(OH)_2D_3$; 22-oxa-calcitriol	Upregulation of p21 and p27; Downregulation of cyclins (D1, E and A), CDK2 and CDK4 [26]
AsPc-1; Bx-PC3; T3M-4	EB1089; CB1093	Unclear [27]
GER	EB1089	Unclear [28]
(Blood cancers)		
HL-60	EB1089 + TGF-β	Upregulation of p27 protein expression [29]
(Liver cancer)		
HepG2	$1\alpha,25(OH)_2D_3$, EB1089, CB1093	Unclear [30]

(Squamous cell carcinoma)		
SCC (mouse)	1α,25$(OH)_2D_3$	Induction of p27; inhibition of p21 [31]
SCC25	1α,25$(OH)_2D_3$, EB1089	Upregulation of GADD45α and induction of CDKI p21$^{waf1/cip1}$, leading to arrest of cell proliferation at the G0/G1 phase [32]
AT-84 (mouse)	EB1089, 1α,25$(OH)_2D_3$	Inhibition of p21$^{WAF1/CIP1}$ expression and induction of expression of p27^{KIP1} protein; upregulation of gadd45α expression [33]
**2. Apoptosis**		
(Breast cancer)		
MCF-7	1α,25$(OH)_2D_3$	Downregulation of survivin through autocrine TGF-β signaling and p38 MAPK activation [34]
	1α,25$(OH)_2D_3$	Disruption of mitochondrial function associated with Bax translocation to mitochondria, cytochrome c release, and production of ROS [35]
	1α,25$(OH)_2D_3$ or EB1089	Reduction of Bcl-2 and induction of p53 protein; induction of TRPM-2 (clusterin) mRNA, a gene associated with onset of apoptosis [36]
	EB1089 + adriamycin	Inhibition of the increase in p21waf1/cip1 levels induced by adriamycin and interferes with induction of MAP kinase activity by ionizing radiation [37]
	1α,25$(OH)_2D_3$ +4-hydroxytamoxifen	Activation of vitamin-D-mediated signalling or disruption of estrogen-dependent signalling [38]
	1α,25$(OH)_2D_3$	Upregulation of cyclin G1 and cyclin I, PAK-1, p53, Rb2 (p130), IGFBP5 and caspases [39]
	ILX 23-7553 + radiation	Unclear [40, 41]
	ILX 23-7553 + adriamycin	Unclear [41]
ER+ MCF-7	1α,25$(OH)_2D_3$; MART-10	Induction of BAX/Bcl expression, and subsequent cytochrome C release from mitochondria to cytosol [42]
(Prostate cancer)		
LNCaP	1α,25$(OH)_2D_3$	Downregulation of anti-apoptotic Bcl-2 and Bcl-X_L proteins [43]
PC3	1α,25$(OH)_2D_3$ + Paclitaxel	Reduction of p21^{Waf-1} expression [44]
Colorectal cancer		
HT-29; SW620	1α,25$(OH)_2D_3$; EB1089	Upregulation of the pro-apoptotic BAK protein expression [45]
(Ovarian cancer)		
OVCAR3	1α,25$(OH)_2D_3$	Reduction of *hTERT* mRNA and downregulation of telomerase activity [46]
(Blood cancers)		
NB-4; HL-60	Paricalcitol + As_2O_3	Induction of monocytic differentiation; downregulation of Bcl-2 and Bcl-x_L [47]

B-CLL	EB1089	Activation of p38 MAP kinase and suppression of ERK activity [48]
(Squamous cell carcinoma)		
SCC (mouse)	$1\alpha,25(OH)_2D_3$ + dexamethasone	Increased VDR protein levels, GR protein levels and ligand binding; reduction of phosphor-Erk1/2 and phosphor-Akt levels [49]
	$1\alpha,25(OH)_2D_3$ + cisplatin	Upregulation of MEKK-1 [50]
**3. Differentiation**		
(Colorectal cancer)		
SW480-ADH	$1\alpha,25(OH)_2D_3$	Induction of E-cadherin and repression of β-catenin–TCF-4 transcriptional activity [51]
	$1\alpha,25(OH)_2D_3$	Upregulation of DKK-1 RNA and protein expression and thus of Wnt–β-catenin-signaling pathway [52]
CaCo-2	$1\alpha,25(OH)_2D_3$	Induction of *c-jun* mRNA and protein expression; activation of ERK2 and JNK1; stimulation of DNA binding and transcriptional activity of AP-1 by ERK- and JNK-dependent mechanisms [53]
(Blood cancers)		
HL-60	$1\alpha,25(OH)_2D_3$	Induction of phagocytosis and nonspecific acid esterase activity; reduction of NBT [54]
	Gemini-19-nor	Induction of CD11b and CD14 expression; induction of $p27^{kip1}$ and PTEN expression [55]
NB-4	$1\alpha,25(OH)_2D_3$ + bryostatin-1	Stimulation of ALP expression and further promote appearance of monocyte/macrophage-like cells [56]
U937	$1\alpha,25(OH)_2D_3$	Ligand-modulated transcriptional induction of the p21 gene [57]
(Prostate cancer)		
LNCaP	$1\alpha,25(OH)_2D_3$<br>$1,24\text{-}(OH)_2D_2$	Increased PSA levels; induction of AR mRNA and protein expression [58]
**4. Inhibition of invasion and metastasis**		
(Breast cancer)		
MDA-MB-231	$1\alpha,25(OH)_2D_3$; CB 1093	Inhibition of expression and activity of cell invasion-associated serine proteases and metalloproteinases and induction of their inhibitors [59]
(Pancreatic cancer)		
BxPC-3; PANC	$1\alpha,25(OH)_2D_3$; MART-10	Downregulation of Snail, Slug, and Vimentin expression; decreased MMP-2 and -9 secretion [60]
(Prostate cancer)		
LNCaP	$1\alpha,25(OH)_2D_3$	Upregulation of IGFBP3 [61]
**5. Anti-angiogenesis**		
(Colorectal cancer)		
SW480-ADH	$1\alpha,25(OH)_2D_3$	Induction of VEGF and the potent antiangiogenic factor *TSP-1* expression [21]
(Prostate cancer)		

LNCaP	$1\alpha,25(OH)_2D_3$	Suppression of NF-κB signal and IL-8 secretion at mRNA and protein levels; inhibition of human PCa cell-induced endothelial cell migration and tube formation [62]
TDEC/SCC	$1\alpha,25(OH)_2D_3$	Inhibition of the growth of TDECs and normal endothelial cells; increased VDR and $p27^{Kip1}$ protein levels; reduction of $p21^{Waf1}$, phospho-ERK1/2 and phospho-Akt levels leading to the hypophosphorylation of Rb in $TDEC_{SCC}$ [63]
RWPE1	$1\alpha,25(OH)_2D_3$	Suppression of WNT, Notch, NF-κB, and IGF1 signaling [64]

15-PGDH, 15-hydroxyprostaglandin dehydrogenase; As_2O_3, arsenic trioxide; ALP, alkaline phosphatase; BAX, Bcl-2-associated X-protein; BRCA, breast cancer susceptibility gene; Bcl-2, B-cell lymphoma 2; c-MYC, cellular Myelocytomatosis; CDK, cyclin-dependent kinase; COX-2, cyclooxygenase-2; EB1089, seocalcitol, 1-dihydroxy-22,24-diene-24,26,27-trihomo-vitamin D_3; EGFR, epidermal growth factor receptor; ERK, extracellular signal–regulated kinase; GADD45α, growth arrest and DNA-damageinducible 45, alpha; Gemini-19-nor, 21-(3-methyl-hydroxy-butyl)-19-nor-D_3; HDAC1, Histone deacetylase 1; hTERT, human telomerase reverse transcriptase; HUVEC, human umbilical vein endothelial cells; IGFBP5, insulin-like growth factor binding protein-5; IGF-IIR, insulin-like growth factor II receptors; MAD1, mitotic arrest deficient protein 1; MAPK, mitogen-activated protein kinase; MEKK1, mitogen-activated protein kinase kinase kinase 1; NSAIDs, non-steroidal anti-inflammatory drugs; OCT, maxacalcitol, 22-oxa-$1\alpha,25(OH)_2D_3$; PAK-1, p21-activated kinase-1; PTP, Protein tyrosine phosphatases; paricalcitol, 19-nor-$1,25(OH)_2D_2$; PGE2, Prostaglandin E2; PTEN, Phosphatase and tensin homolog; PSA, prostate specific antigen; RA, retinoic acid; Rb, retinoblastoma protein; ROS, reactive oxygen species; TCF4, transcription factor 4; TDEC/SCC: tumour-derived endothelial cells from SCC cell line; TGF-β, Transforming growth factor beta; TRPM2, transient receptor potential cation channel subfamily m member 2; TSA, trichostatin A; TSP-1, thrombospondin-1; VDR, vitamin D receptor; VEGF, vascular endothelial growth factor.

References

1. Salehi-Tabar, R.; Nguyen-Yamamoto, L.; Tavera-Mendoza, L.E.; Quail, T.; Dimitrov, V.; An, B.S.; Glass, L.; Goltzman, D.; White, J.H. Vitamin D receptor as a master regulator of the c-MYC/MXD1 network. *Proc. Natl. Acad. Sci. USA* **2012,** *109*, 18827-18832.
2. Jensen, S.S.; Madsen, M.W.; Lukas, J.; Binderup, L.; Bartek, J. Inhibitory effects of 1 alpha,25-dihydroxyvitamin D-3 on the G(1)-S phase-controlling machinery. *Mol. Endocrinol.* **2001,** *15*, 1370-1380.
3. Campbell, M.J.; Gombart, A.F.; Kwok, S.H.; Park, S.; Koeffler, H.P. The anti-proliferative effects of 1 alpha,25(OH)(2)D-3 on breast and prostate cancer cells are associated with induction of BRCA1 gene expression. *Oncogene* **2000,** *19*, 5091-5097.
4. Capiati, D.A.; Rossi, A.M.; Picotto, G.; Benassati, S.; Boland, R.L. Inhibition of serum-stimulated mitogen activated protein kinase by 1 alpha,25(OH)(2)-vitamin D-3 in MCF-7 breast cancer cells. *J. Cell. Biochem.* **2004,** *93*, 384-397.
5. Koga, M.; Eisman, J.A.; Sutherland, R.L. Regulation of epidermal growth factor receptor levels by 1,25-dihydroxyvitamin D3 in human breast cancer cells. *Cancer Res.* **1988,** *48*, 2734-2739.
6. Huang, Y.C.; Hung, W.C. 1,25-dihydroxyvitamin D3 transcriptionally represses p45Skp2 expression via the Sp1 sites in human prostate cancer cells. *J. Cell. Physiol.* **2006,** *209*, 363-369.

7. Yang, E.S.; Burnstein, K.L. Vitamin D inhibits G1 to S progression in LNCaP prostate cancer cells through p27Kip1 stabilization and Cdk2 mislocalization to the cytoplasm. *J. Biol. Chem.* **2003,** *278*, 46862-46868.
8. Rao, A.; Coan, A.; Welsh, J.E.; Barclay, W.W.; Koumenis, C.; Cramer, S.D. Vitamin D receptor and p21/WAF1 are targets of genistein and 1,25-dihydroxyvitamin D3 in human prostate cancer cells. *Cancer Res.* **2004,** *64*, 2143-2147.
9. Swami, S.; Krishnan, A.V.; Moreno, J.; Bhattacharyya, R.B.; Peehl, D.M.; Feldman, D. Calcitriol and genistein actions to inhibit the prostaglandin pathway: potential combination therapy to treat prostate cancer. *J. Nutr.* **2007,** *137*, 205S-210S.
10. Qiao, S.; Tuohimaa, P. Vitamin D3 inhibits fatty acid synthase expression by stimulating the expression of long-chain fatty-acid-CoA ligase 3 in prostate cancer cells. *FEBS Lett.* **2004,** *577*, 451-454.
11. Ikeda, N.; Uemura, H.; Ishiguro, H.; Hori, M.; Hosaka, M.; Kyo, S.; Miyamoto, K.; Takeda, E.; Kubota, Y. Combination treatment with 1alpha,25-dihydroxyvitamin D3 and 9-cis-retinoic acid directly inhibits human telomerase reverse transcriptase transcription in prostate cancer cells. *Mol. Cancer Ther.* **2003,** *2*, 739-746.
12. Moreno, J.; Krishnan, A.V.; Swami, S.; Nonn, L.; Peehl, D.M.; Feldman, D. Regulation of prostaglandin metabolism by calcitriol attenuates growth stimulation in prostate cancer cells. *Cancer Res.* **2005,** *65*, 7917-7925.
13. Campbell, M.J.; Elstner, E.; Holden, S.; Uskokovic, M.; Koeffler, H.P. Inhibition of proliferation of prostate cancer cells by a 19-nor-hexafluoride vitamin D3 analogue involves the induction of p21waf1, p27kip1 and E-cadherin. *J. Mol. Endocrinol.* **1997,** *19*, 15-27.
14. Moffatt, K.A.; Johannes, W.U.; Hedlund, T.E.; Miller, G.J. Growth inhibitory effects of 1alpha, 25-dihydroxyvitamin D(3) are mediated by increased levels of p21 in the prostatic carcinoma cell line ALVA-31. *Cancer Res.* **2001,** *61*, 7122-7129.
15. Gommersall, L.M.; Khanim, F.L.; Peehl, D.M.; Doherty, A.H.; Campbell, M.J. Epigenetic repression of transcription by the Vitamin D3 receptor in prostate cancer cells. *J. Steroid Biochem. Mol. Biol.* **2004,** *89-90*, 251-256.
16. Washington, M.N.; Kim, J.S.; Weigel, N.L. 1alpha,25-dihydroxyvitamin D3 inhibits C4-2 prostate cancer cell growth via a retinoblastoma protein (Rb)-independent G1 arrest. *Prostate* **2011,** *71*, 98-110.
17. Bao, B.Y.; Hu, Y.C.; Ting, H.J.; Lee, Y.F. Androgen signaling is required for the vitamin D-mediated growth inhibition in human prostate cancer cells. *Oncogene* **2004,** *23*, 3350-3360.
18. Tong, W.M.; Hofer, H.; Ellinger, A.; Peterlik, M.; Cross, H.S. Mechanism of antimitogenic action of vitamin D in human colon carcinoma cells: Relevance for suppression of epidermal growth factor-stimulated cell growth. *Oncol. Res.* **1999,** *11*, 77-84.
19. Chen, A.P.; Davis, B.H.; Sitrin, M.D.; Brasitus, T.A.; Bissonnette, M. Transforming growth factor-beta 1 signaling contributes to Caco-2 cell growth inhibition induced by 1,25(OH)(2)D-3. *American Journal of Physiology-Gastrointestinal and Liver Physiology* **2002,** *283*, G864-G874.
20. Gaschott, T.; Stein, J. Short-chain fatty acids and colon cancer cells: The vitamin D receptor - Butyrate connection. *Vitamin D Analogs in Cancer Prevention and Therapy* **2003,** *164*, 247-257.
21. Fernandez-Garcia, N.I.; Palmer, H.G.; Garcia, M.; Gonzalez-Martin, A.; del Rio, M.; Barettino, D.; Volpert, O.; Munoz, A.; Jimenez, B. 1alpha,25-Dihydroxyvitamin D3 regulates the expression of Id1 and Id2 genes and the angiogenic phenotype of human colon carcinoma cells. *Oncogene* **2005,** *24*, 6533-6544.
22. Oh, Y.S.; Kim, E.J.; Schaffer, B.S.; Kang, Y.H.; Binderup, L.; MacDonald, R.G.; Park, J.H. Synthetic low-calcaemic vitamin D(3) analogues inhibit secretion of insulin-like growth factor II and stimulate production of insulin-like growth factor-binding protein-6 in conjunction with growth suppression of HT-29 colon cancer cells. *Mol. Cell. Endocrinol.* **2001,** *183*, 141-149.

23. Meyer, M.B.; Goetsch, P.D.; Pike, J.W. VDR/RXR and TCF4/beta-catenin cistromes in colonic cells of colorectal tumor origin: impact on c-FOS and c-MYC gene expression. *Mol. Endocrinol.* **2012,** *26*, 37-51.
24. Li, P.F.; Li, C.R.; Zhao, X.H.; Zhang, X.H.; Nicosia, S.V.; Bai, W.L. P27(Kip1) stabilization and G(1) arrest by 1,25-dihydroxyvitamin D-3 in ovarian cancer cells mediated through down-regulation of cyclin E/cyclin-dependent kinase 2 and Skp1-Cullin-F-box Protein/Skp2 ubiquitin ligase. *J. Biol. Chem.* **2004,** *279*, 25260-25267.
25. Jiang, F.; Li, P.F.; Fornace, A.J.; Nicosia, S.V.; Bai, W.L. G(2)/M arrest by 1,25-dihydroxyvitamin D-3 in ovarian cancer cells mediated through the induction of GADD45 via an exonic enhancer. *J. Biol. Chem.* **2003,** *278*, 48030-48040.
26. Kawa, S.; Nikaido, T.; Aoki, Y.; Zhai, Y.; Kumagai, T.; Furihata, K.; Fujii, S.; Kiyosawa, K. Vitamin D analogues up-regulate p21 and p27 during growth inhibition of pancreatic cancer cell lines. *Br. J. Cancer* **1997,** *76*, 884-889.
27. Zugmaier, G.; Jager, R.; Grage, B.; Gottardis, M.M.; Havemann, K.; Knabbe, C. Growth-inhibitory effects of vitamin D analogues and retinoids on human pancreatic cancer cells. *Br. J. Cancer* **1996,** *73*, 1341-1346.
28. Colston, K.W.; James, S.Y.; OforiKuragu, E.A.; Binderup, L.; Grant, A.G. Vitamin D receptors and anti-proliferative effects of vitamin D derivatives in human pancreatic carcinoma cells in vivo and in vitro. *Br. J. Cancer* **1997,** *76*, 1017-1020.
29. Jung, C.W.; Kim, E.S.; Seol, J.G.; Park, W.H.; Lee, S.J.; Kim, B.K.; Lee, Y.Y. Antiproliferative effect of a vitamin D3 analog, EB1089, on HL-60 cells by the induction of TGF-beta receptor. *Leuk. Res.* **1999,** *23*, 1105-1112.
30. Akhter, J.; Lu, Y.; Finlay, I.; Pourgholami, M.H.; Morris, D.L. 1alpha,25-Dihydroxyvitamin D3 and its analogues, EB1089 and CB1093, profoundly inhibit the in vitro proliferation of the human hepatoblastoma cell line HepG2. *ANZ J. Surg.* **2001,** *71*, 414-417.
31. Hershberger, P.A.; Modzelewski, R.A.; Shurin, Z.R.; Rueger, R.M.; Trump, D.L.; Johnson, C.S. 1,25-Dihydroxycholecalciferol (1,25-D3) inhibits the growth of squamous cell carcinoma and down-modulates p21(Waf1/Cip1) in vitro and in vivo. *Cancer Res.* **1999,** *59*, 2644-2649.
32. Akutsu, N.; Lin, R.; Bastien, Y.; Bestawros, A.; Enepekides, D.J.; Black, M.J.; White, J.H. Regulation of gene Expression by 1alpha,25-dihydroxyvitamin D3 and Its analog EB1089 under growth-inhibitory conditions in squamous carcinoma Cells. *Mol. Endocrinol.* **2001,** *15*, 1127-1139.
33. Prudencio, J.; Akutsu, N.; Benlimame, N.; Wang, T.; Bastien, Y.; Lin, R.; Black, M.J.; Alaoui-Jamali, M.A.; White, J.H. Action of low calcemic 1alpha,25-dihydroxyvitamin D3 analogue EB1089 in head and neck squamous cell carcinoma. *J. Natl. Cancer Inst.* **2001,** *93*, 745-753.
34. Li, F.; Ling, X.; Huang, H.; Brattain, L.; Apontes, P.; Wu, J.; Binderup, L.; Brattain, M.G. Differential regulation of survivin expression and apoptosis by vitamin D3 compounds in two isogenic MCF-7 breast cancer cell sublines. *Oncogene* **2005,** *24*, 1385-1395.
35. Narvaez, C.J.; Welsh, J. Role of mitochondria and caspases in vitamin D-mediated apoptosis of MCF-7 breast cancer cells. *J. Biol. Chem.* **2001,** *276*, 9101-9107.
36. James, S.Y.; Mackay, A.G.; Colston, K.W. Effects of 1,25 dihydroxyvitamin D3 and its analogues on induction of apoptosis in breast cancer cells. *J. Steroid Biochem. Mol. Biol.* **1996,** *58*, 395-401.
37. Sundaram, S.; Chaudhry, M.; Reardon, D.; Gupta, M.; Gewirtz, D.A. The vitamin D3 analog EB 1089 enhances the antiproliferative and apoptotic effects of adriamycin in MCF-7 breast tumor cells. *Breast Cancer Res. Treat.* **2000,** *63*, 1-10.
38. Welsh, J. Induction of apoptosis in breast cancer cells in response to vitamin D and antiestrogens. *Biochem. Cell Biol.* **1994,** *72*, 537-545.
39. Swami, S.; Raghavachari, N.; Muller, U.R.; Bao, Y.P.; Feldman, D. Vitamin D growth inhibition of breast cancer cells: gene expression patterns assessed by cDNA microarray. *Breast Cancer Res. Treat.* **2003,** *80*, 49-62.

40. Polar, M.K.; Gennings, C.; Park, M.; Gupta, M.S.; Gewirtz, D.A. Effect of the vitamin D3 analog ILX 23-7553 on apoptosis and sensitivity to fractionated radiation in breast tumor cells and normal human fibroblasts. *Cancer Chemother. Pharmacol.* **2003,** *51*, 415-421.
41. Chaudhry, M.; Sundaram, S.; Gennings, C.; Carter, H.; Gewirtz, D.A. The vitamin D3 analog, ILX-23-7553, enhances the response to adriamycin and irradiation in MCF-7 breast tumor cells. *Cancer Chemother. Pharmacol.* **2001,** *47*, 429-436.
42. Chiang, K.C.; Yeh, C.N.; Chen, S.C.; Shen, S.C.; Hsu, J.T.; Yeh, T.S.; Pang, J.H.; Su, L.J.; Takano, M.; Kittaka, A.; Juang, H.H.; Chen, T.C. MART-10, a New Generation of Vitamin D Analog, Is More Potent than 1alpha,25-Dihydroxyvitamin D(3) in Inhibiting Cell Proliferation and Inducing Apoptosis in ER+ MCF-7 Breast Cancer Cells. *Evid. Based Complement. Alternat. Med.* **2012,** *2012*, 310872.
43. Blutt, S.E.; McDonnell, T.J.; Polek, T.C.; Weigel, N.L. Calcitriol-induced apoptosis in LNCaP cells is blocked by overexpression of bcl-2. *Endocrinology* **2000,** *141*, 10-17.
44. Hershberger, P.A.; Yu, W.D.; Modzelewski, R.A.; Rueger, R.M.; Johnson, C.S.; Trump, D.L. Calcitriol (1,25-dihydroxycholecalciferol) enhances paclitaxel antitumor activity in vitro and in vivo and accelerates paclitaxel-induced apoptosis. *Clin. Cancer. Res.* **2001,** *7*, 1043-1051.
45. Diaz, G.D.; Paraskeva, C.; Thomas, M.G.; Binderup, L.; Hague, A. Apoptosis is induced by the active metabolite of vitamin D-3 and its analogue EB1089 in colorectal adenoma and carcinoma cells: Possible implications for prevention and therapy. *Cancer Res.* **2000,** *60*, 2304-2312.
46. Jiang, F.; Bao, J.Y.; Li, P.F.; Nicosia, S.V.; Bai, W.L. Induction of ovarian cancer cell apoptosis by 1,25-dihydroxyvitamin D-3 through the down-regulation of telomerase. *J. Biol. Chem.* **2004,** *279*, 53213-53221.
47. Kumagai, T.; Shih, L.Y.; Hughes, S.V.; Desmond, J.C.; O'Kelly, J.; Hewison, M.; Koeffler, H.P. 19-Nor-1,25(OH)(2)D-2 (a novel, noncalcemic vitamin D analogue), combined with arsenic trioxide, has potent antitumor activity against myeloid leukemia. *Cancer Res.* **2005,** *65*, 2488-2497.
48. Pepper, C.; Thomas, A.; Hoy, T.; Milligan, D.; Bentley, P.; Fegan, C. The vitamin D3 analog EB1089 induces apoptosis via a p53-independent mechanism involving p38 MAP kinase activation and suppression of ERK activity in B-cell chronic lymphocytic leukemia cells in vitro. *Blood* **2003,** *101*, 2454-2460.
49. Bernardi, R.J.; Trump, D.L.; Yu, W.D.; McGuire, T.F.; Hershberger, P.A.; Johnson, C.S. Combination of 1 alpha,25-dihydroxyvitamin D-3 with dexamethasone enhances cell cycle arrest and apoptosis: Role of nuclear receptor cross-talk and Erk/Akt signaling. *Clin. Cancer. Res.* **2001,** *7*, 4164-4173.
50. Hershberger, P.A.; McGuire, T.F.; Yu, W.D.; Zuhowski, E.G.; Schellens, J.H.; Egorin, M.J.; Trump, D.L.; Johnson, C.S. Cisplatin potentiates 1,25-dihydroxyvitamin D3-induced apoptosis in association with increased mitogen-activated protein kinase kinase kinase 1 (MEKK-1) expression. *Mol. Cancer Ther.* **2002,** *1*, 821-829.
51. Palmer, H.G.; Gonzalez-Sancho, J.M.; Espada, J.; Berciano, M.T.; Puig, I.; Baulida, J.; Quintanilla, M.; Cano, A.; de Herreros, A.G.; Lafarga, M.; Munoz, A. Vitamin D(3) promotes the differentiation of colon carcinoma cells by the induction of E-cadherin and the inhibition of beta-catenin signaling. *J. Cell Biol.* **2001,** *154*, 369-387.
52. Aguilera, O.; Pena, C.; Garcia, J.M.; Larriba, M.J.; Ordonez-Moran, P.; Navarro, D.; Barbachano, A.; Lopez de Silanes, I.; Ballestar, E.; Fraga, M.F.; Esteller, M.; Gamallo, C.; Bonilla, F.; Gonzalez-Sancho, J.M.; Munoz, A. The Wnt antagonist DICKKOPF-1 gene is induced by 1alpha,25-dihydroxyvitamin D3 associated to the differentiation of human colon cancer cells. *Carcinogenesis* **2007,** *28*, 1877-1884.
53. Chen, A.; Davis, B.H.; Bissonnette, M.; Scaglione-Sewell, B.; Brasitus, T.A. 1,25-Dihydroxyvitamin D(3) stimulates activator protein-1-dependent Caco-2 cell differentiation. *J. Biol. Chem.* **1999,** *274*, 35505-35513.

54. Mangelsdorf, D.J.; Koeffler, H.P.; Donaldson, C.A.; Pike, J.W.; Haussler, M.R. 1,25-Dihydroxyvitamin D3-induced differentiation in a human promyelocytic leukemia cell line (HL-60): receptor-mediated maturation to macrophage-like cells. *J. Cell Biol.* **1984,** *98*, 391-398.
55. Hisatake, J.; O'Kelly, J.; Uskokovic, M.R.; Tomoyasu, S.; Koeffler, H.P. Novel vitamin D-3 analog, 21-(3-methyl-3-hydroxy-butyl)-19-nor D-3, that modulates cell growth, differentiation, apoptosis, cell cycle, and induction of PTEN in leukemic cells. *Blood* **2001,** *97*, 2427-2433.
56. Song, X.D.; Norman, A.W. Bryostatin-1 and 1alpha,25-dihydroxyvitamin D3 synergistically stimulate the differentiation of NB4 acute promyelocytic leukemia cells. *Leukemia* **1999,** *13*, 275-281.
57. Liu, M.; Lee, M.H.; Cohen, M.; Bommakanti, M.; Freedman, L.P. Transcriptional activation of the Cdk inhibitor p21 by vitamin D3 leads to the induced differentiation of the myelomonocytic cell line U937. *Genes Dev.* **1996,** *10*, 142-153.
58. Bauer, J.A.; Thompson, T.A.; Church, D.R.; Ariazi, E.A.; Wilding, G. Growth inhibition and differentiation in human prostate carcinoma cells induced by the vitamin D analog 1alpha,24-dihydroxyvitamin D2. *Prostate* **2003,** *55*, 159-167.
59. Koli, K.; Keski-Oja, J. 1alpha,25-dihydroxyvitamin D3 and its analogues down-regulate cell invasion-associated proteases in cultured malignant cells. *Cell Growth Differ.* **2000,** *11*, 221-229.
60. Chiang, K.C.; Yeh, C.N.; Hsu, J.T.; Jan, Y.Y.; Chen, L.W.; Kuo, S.F.; Takano, M.; Kittaka, A.; Chen, T.C.; Chen, W.T.; Pang, J.H.; Yeh, T.S.; Juang, H.H. The vitamin D analog, MART-10, represses metastasis potential via downregulation of epithelial-mesenchymal transition in pancreatic cancer cells. *Cancer Lett.* **2014,** *354*, 235-244.
61. Krishnan, A.V.; Shinghal, R.; Raghavachari, N.; Brooks, J.D.; Peehl, D.M.; Feldman, D. Analysis of vitamin D-regulated gene expression in LNCaP human prostate cancer cells using cDNA microarrays. *Prostate* **2004,** *59*, 243-251.
62. Bao, B.Y.; Yao, J.; Lee, Y.F. 1alpha, 25-dihydroxyvitamin D3 suppresses interleukin-8-mediated prostate cancer cell angiogenesis. *Carcinogenesis* **2006,** *27*, 1883-1893.
63. Bernardi, R.J.; Johnson, C.S.; Modzelewski, R.A.; Trump, D.L. Antiproliferative effects of 1alpha,25-dihydroxyvitamin D(3) and vitamin D analogs on tumor-derived endothelial cells. *Endocrinology* **2002,** *143*, 2508-2514.
64. Kovalenko, P.L.; Zhang, Z.; Cui, M.; Clinton, S.K.; Fleet, J.C. 1,25 dihydroxyvitamin D-mediated orchestration of anticancer, transcript-level effects in the immortalized, non-transformed prostate epithelial cell line, RWPE1. *BMC Genomics* **2010,** *11*, 26.
